# Neuro-immune-bone axis in post-traumatic bone regeneration: temporal regulation, dysregulation mechanisms, and therapeutic implications

**DOI:** 10.3389/fimmu.2026.1911131

**Published:** 2026-09-22

**Authors:** Zhonghua Wang, Haijun Zhang

**Affiliations:** Department of Orthopedics, Gansu Provincial Second People’s Hospital, Lanzhou, Gansu, China

**Keywords:** fracture healing, immune regulation, nerve reinnervation, neuro-immune-bone axis, neurovascular coupling, nonunion, post-traumatic bone regeneration

## Abstract

Post-traumatic bone regeneration extends beyond a mere osteogenic event to encompass a dynamic reparative cascade characterized by the coordinated interplay of inflammation, nerve reinnervation, angiogenesis, mesenchymal stem cell differentiation, and osteoblast-osteoclast coupling. While immune and neural regulations have been independently implicated in bone repair, a unified mechanistic framework and sufficient human evidence regarding the spatiotemporal synergy of neural signals with immune cells-and the extent to which this interaction dictates healing outcomes-remain elusive. Current research suggests that sensory nerves, sympathetic nerves, and their released neuropeptides and neurotransmitters can engage in bidirectional communication with immune cells such as macrophages, T cells, and neutrophils, providing a new conceptual framework for integrating injury perception, inflammatory transition, neurovascular reconstruction, and bone remodeling from the perspective of the “neuro-immune-bone axis”. This review examines the temporal dynamics of the immune response and nerve reinnervation during post-traumatic bone repair. It places particular emphasis on the bidirectional interactions between specific neural signals and immune cells, exploring how downstream signaling nodes transduce neural inputs into altered immune states that subsequently influence BMSCs, osteoblasts, angiogenesis, and bone remodeling. Additionally, this article reviews the potential links between abnormal neuro-immune interactions and delayed healing, nonunion, and chronic pain, and discusses the possible alterations of this regulatory network under conditions such as diabetes, osteoporosis, and aging, along with their therapeutic implications. Notably, the evidentiary strength in this field varies considerably: many mechanistic insights are derived from animal models, *in vitro* studies, or non-fracture tissue injury models, and the effects of certain neuropeptides, sympathetic signals, and immune subsets exhibit significant stage, dose, and tissue-context dependence. Regarding the causal contribution of the neuro-immune axis to human fracture healing and whether targeted interventions can improve clinical bone healing outcomes, long-term longitudinal studies and high-quality clinical validation remain lacking. Therefore, at this stage, it is more appropriate to regard the neuro-immune-bone axis as an integrative working framework for explaining multi-system synergistic repair after trauma, rather than a fully established linear causal pathway. Future research should combine temporal, multicellular, and spatial evidence to identify key regulatory nodes at different repair stages and evaluate their feasibility as stage-specific therapeutic targets.

## 1 Introduction

Bone regeneration after orthopedic trauma is a complex and highly ordered biological process, the success of which is directly related to the patient’s functional recovery and quality of life (1). Although techniques for fracture and bone defect repair continue to advance, delayed healing, nonunion, and large segmental bone defects remain significant challenges in clinical treatment, particularly under complex pathological conditions such as severe soft tissue injury, infection, diabetes, osteoporosis, and aging. Such healing impairments not only prolong the treatment cycle and increase the risk of reoperation but also impose a heavy burden on patients and the healthcare system (2–4). Therefore, a deeper understanding of the regulatory mechanisms underlying post-traumatic bone regeneration and the exploration of new strategies to improve the quality of complex fracture and bone defect repair remain important directions in the field of orthopedic regenerative medicine.

Traditionally, bone regeneration research has predominantly centered on local factors, including osteoblasts, osteoclasts, bone marrow mesenchymal stem cells, angiogenesis, growth factors, and bone repair materials, whereas the systemic regulatory roles of the nervous and immune systems in bone repair have garnered comparatively less attention (5, 6). In recent years, interdisciplinary studies in osteoimmunology, neuroimmunology, and bone biology have shown that post-traumatic bone repair is essentially a tissue regeneration process involving the coordinated participation of inflammation, nerves, blood vessels, and bone cells (7). However, existing studies have mostly focused separately on dual-system interactions, such as bone-immune, nerve-bone, or neurovascular crosstalk, and there remains a lack of systematic integration regarding how neural signals, immune state transitions, neurovascular reconstruction, and bone cell responses form a coordinated network across different repair stages (8). Specifically, questions regarding the synchronization of nerve reinnervation and inflammation resolution, the manner in which neural signals reshape immune function, and the reciprocal influence of immune cells on nerve regeneration and sensitization remain supported only by fragmented evidence, characterized by significant variability in evidentiary strength across these mechanisms.

Bone tissue is richly innervated by sensory and sympathetic nerves. Neuropeptides released from nerve terminals, including calcitonin gene-related peptide (CGRP), substance P (SP), and norepinephrine (NE), modulate the functions of immune cells, vascular endothelial cells, and bone cells (9, 10). Conversely, cytokines, chemokines, and neurotrophic factors secreted by immune cells, such as macrophages, T cells, and neutrophils-reciprocally regulate nerve regeneration, neural sensitization, and the local repair microenvironment (11, 12). Based on this cross-system connection, this review adopts the “neuro-immune-bone axis” as an integrative working framework. This framework is not intended to supersede existing concepts; rather, it complements them. While osteoimmunology focuses on immune-bone cell interactions, nerve-bone research emphasizes neural regulation of bone metabolism, and neurovascular coupling addresses the coordinated reconstruction of nerves and vessels, the “neuro-immune-bone axis” integrates these processes within the shared spatiotemporal context of wound repair. Specifically, it elucidates how these systems collectively link inflammation initiation and resolution, nerve reinnervation, angiogenesis, osteoblast-osteoclast coupling, and callus remodeling via shared cellular and molecular nodes. Therefore, this concept is more suitable as a working framework for explaining multi-system synergy and mismatch, rather than an established linear causal pathway.

Within this framework, this review first outlines the immune temporal sequence and macrophage state changes during fracture healing, and discusses bone tissue innervation, post-traumatic reinnervation, and neurovascular reconstruction; it then focuses on the bidirectional communication between neuropeptides, sympathetic nerve signals, and immune cells, as well as key downstream nodes such as NF-κB, MAPK, and JAK/STAT, and the potential effects of these interactions on osteogenesis, osteoclastogenesis, angiogenesis, and bone remodeling. On this basis, it further discusses network imbalances in the context of delayed healing, nonunion, chronic pain, diabetes, osteoporosis, and aging, and evaluates corresponding intervention strategies and their translational limitations. This review aims to integrate existing evidence through a temporal and cross-system perspective, while clarifying issues such as model dependence, insufficient causal evidence, and limited clinical validation, thereby providing a conceptual basis for future mechanistic research and stage-specific interventions.

## 2 The basic process of post-traumatic bone regeneration and the immune timeline

### 2.1 Stages of post-traumatic bone repair

Fracture healing typically progresses through continuous and overlapping stages, including hematoma formation and inflammatory initiation, cartilaginous callus formation, hard callus formation, and bone remodeling (13, 14). Different stages are characterized not only by changes in tissue structure and mechanical function but also by dynamic remodeling of immune cell composition, neural activity, angiogenesis, and bone cell function (15). Therefore, post-traumatic bone regeneration is better understood as a continuous process in which neural, immune, vascular, and bone repair programs are temporally interconnected, rather than a simple osteogenic sequence.

During the hematoma formation and inflammatory initiation stage, local vascular rupture forms a hematoma rich in platelets, fibrin, inflammatory mediators, and growth factors, providing an initial microenvironment for subsequent cell recruitment (16, 17). Subsequently, neutrophils and monocytes/macrophages rapidly enter the injured area, clearing necrotic tissue and releasing mediators such as TNF-α, IL-1β, IL-6, and chemokines (18). Meanwhile, local nerve injury caused by the fracture and activation of sensory nerve endings can be accompanied by changes in the release of neuropeptides such as SP and CGRP, thereby affecting vascular permeability, immune cell recruitment, and early neurogenic inflammation (19). Sympathetic nerve signals may also participate in blood flow and immune regulation during this stage (20). Therefore, the key to the early stage is not simply enhancing inflammation, but forming a local environment sufficient for injury clearance while being able to promptly enter inflammatory resolution.

As inflammation gradually subsides, repair enters the cartilaginous callus formation stage. Mesenchymal stem cells and periosteum-derived osteoprogenitor cells are recruited, proliferate, and undergo chondrogenic differentiation, gradually forming a cartilaginous callus connecting the fracture ends (21, 22). During this period, the immune environment gradually shifts from inflammatory initiation to inflammatory resolution and tissue repair, with pro-angiogenic, matrix remodeling, and repair-supporting functional programs in macrophages gradually increasing (23). Meanwhile, sensory nerve fibers can regrow toward the callus and adjacent areas of neovascularization, suggesting potential synergy between nerve reinnervation and angiogenesis, progenitor cell recruitment, and local immune status (24).

During the hard callus formation stage, the cartilaginous callus gradually mineralizes and is replaced by vascular-rich woven bone, with structural continuity of the fracture ends gradually restored (25). The main features of this stage further shift from inflammatory control to vascular maturation, enhanced osteogenesis, and tissue structural reconstruction. The spatial proximity of nerve fibers and neovascularization within the callus, as well as the regulatory effects of neural signals such as CGRP, SP, and NE on endothelial cells, bone cells, and immune cells, suggests that neural-vascular-immune synergy may participate in maintaining the osteogenic microenvironment (26).

Upon entering the bone remodeling stage, osteoclast-mediated bone resorption and osteoblast-mediated bone formation remain continuously coupled, gradually converting woven bone into lamellar bone with more mature structure and mechanical properties, while restoring the medullary cavity and local bone structure (27, 28). In this phase, acute inflammation gradually subsides, immune cells are more involved in bone metabolism and tissue homeostasis regulation, and neural signals gradually shift from early injury perception and neurogenic inflammation to long-term regulation of blood flow, osteoblast-osteoclast balance, and the local metabolic environment (29). Persistent neuroinflammation or excessive sympathetic activation may disrupt this balance, but direct evidence in human late-stage fracture remodeling remains limited.

Overall, each stage of fracture repair is not dominated sequentially and independently by different cells, but rather accompanied by continuous and overlapping changes in neural, immune, and vascular programs: the early stage is dominated by injury perception, inflammatory initiation, and tissue clearance; the middle stage gradually shifts toward inflammatory resolution, neurovascular reconstruction, and osteogenic support; and the late stage is characterized mainly by osteoblast-osteoclast coupling and restoration of tissue homeostasis (30). This cross-stage coordination may better reflect the overall repair status than changes in a single cell type or a single signal.

### 2.2 Temporal transitions of immune responses and macrophage states

Immune responses span the entire process of post-traumatic bone regeneration; the critical factor lies not in a unidirectional increase or decrease in the number of a specific immune cell type, but in the capacity of different immune functional states to undergo timely transitions according to the repair stage and local demands (31, 32). In the early stage of injury, neutrophils and monocytes/macrophages rapidly infiltrate the fracture hematoma, initiating repair by phagocytosing necrotic tissue and cell debris, and releasing mediators such as TNF-α, IL-1β, IL-6, and chemokines (32, 33). Moderate pro-inflammatory signals help recruit mesenchymal stem cells and osteogenic precursor cells, but excessive or prolonged inflammatory responses may inhibit osteogenesis, promote osteoclastogenesis, and aggravate local tissue damage (34).

The dynamic changes in macrophage functional states are an important component of this immune timeline. In the early stage of injury, functional programs characterized by phagocytic clearance, inflammatory signal production, and cell recruitment are relatively dominant; as repair progresses, functions related to inflammatory resolution, pro-angiogenesis, matrix remodeling, and tissue repair gradually increase, and can support vascularization, callus formation, and bone matrix deposition through factors such as IL-10, TGF-β, and vascular endothelial growth factor (VEGF) (35, 36). Notably, macrophages *in vivo* exhibit high heterogeneity and plasticity, precluding their strict classification into fixed “M1” and “M2” phenotypes. Terms such as pro-inflammatory-like and repair-like states serve best as simplified concepts to describe functional tendencies; the actual fracture microenvironment typically contains multiple continuous, overlapping, and interconvertible cell states, the composition of which is co-determined by the degree of injury, local mechanical environment, blood supply, metabolic status, and other immune signals. Therefore, macrophage changes during fracture healing are better understood as functional state remodeling that occurs with the repair stage: early inflammatory and clearance-related programs are relatively dominant, followed by multiple transitional states, with gradual increases in functional programs related to inflammatory resolution, angiogenesis, matrix remodeling, and tissue repair; these states overlap significantly in time, rather than a strict “M1→M2” unidirectional transition (37). If early inflammatory programs remain persistently dominant, or if inflammatory resolution and repair-supporting functions are inadequately established, neurovascular reconstruction, osteogenic differentiation, and callus maturation may be delayed; conversely, premature suppression of necessary early inflammation may also weaken injury clearance and repair initiation, indicating that macrophage functions are markedly time-window dependent.

Adaptive immune cells may also participate in this temporal regulation. IL-10 and TGF-β signals produced by regulatory T cells (Treg) mediate inflammatory restriction and tissue repair, while Th17 cells and IL-17 signals drive inflammatory amplification and osteoclastogenesis (38, 39). However, simply attributing fracture healing to a “Treg/Th17 balance” may also be an oversimplification. Existing evidence largely comes from osteoimmunology or other inflammatory models, and the direct roles, time windows, and causal contributions of Treg and Th17 in different stages of typical traumatic fractures remain incompletely defined. Therefore, a more cautious understanding is that these T cell subsets may participate in repair by altering the local cytokine environment and osteoblast-osteoclast balance, while their stage-specific contributions still require further validation.

B cells can participate in bone metabolism regulation through pathways such as receptor activator of nuclear factor-κB ligand/osteoprotegerin (RANKL/OPG) and cytokines; however, evidence regarding their dynamic changes and direct functions in traumatic fracture healing is scarcer than for T cells and macrophages, thus they cannot yet be classified as core regulatory cells with defined stage specificity (40). Therefore, the post-traumatic immune timeline is better understood as the continuous remodeling of multiple immune cells and their functional states concurrent with the repair process, rather than being determined by fixed binary patterns such as M1/M2 or Treg/Th17. The temporal remodeling of macrophage functional states may serve as a critical hub connecting early inflammation, inflammatory resolution, neurovascular reconstruction, and subsequent bone formation; however, its stage boundaries, spatial heterogeneity, and synergy with other immune subsets still require further direct spatiotemporal and functional validation (41) (**Figure 1**). To facilitate comparison of immune cell changes across different repair stages, **Table 1** summarizes the relative changes and main roles of major macrophage functional states, Treg, Th17, and other immune cells at each stage of fracture healing; it should be noted that these stages overlap significantly, and especially the stage-specific evidence for Treg and Th17 remains limited.

**Figure 1:**
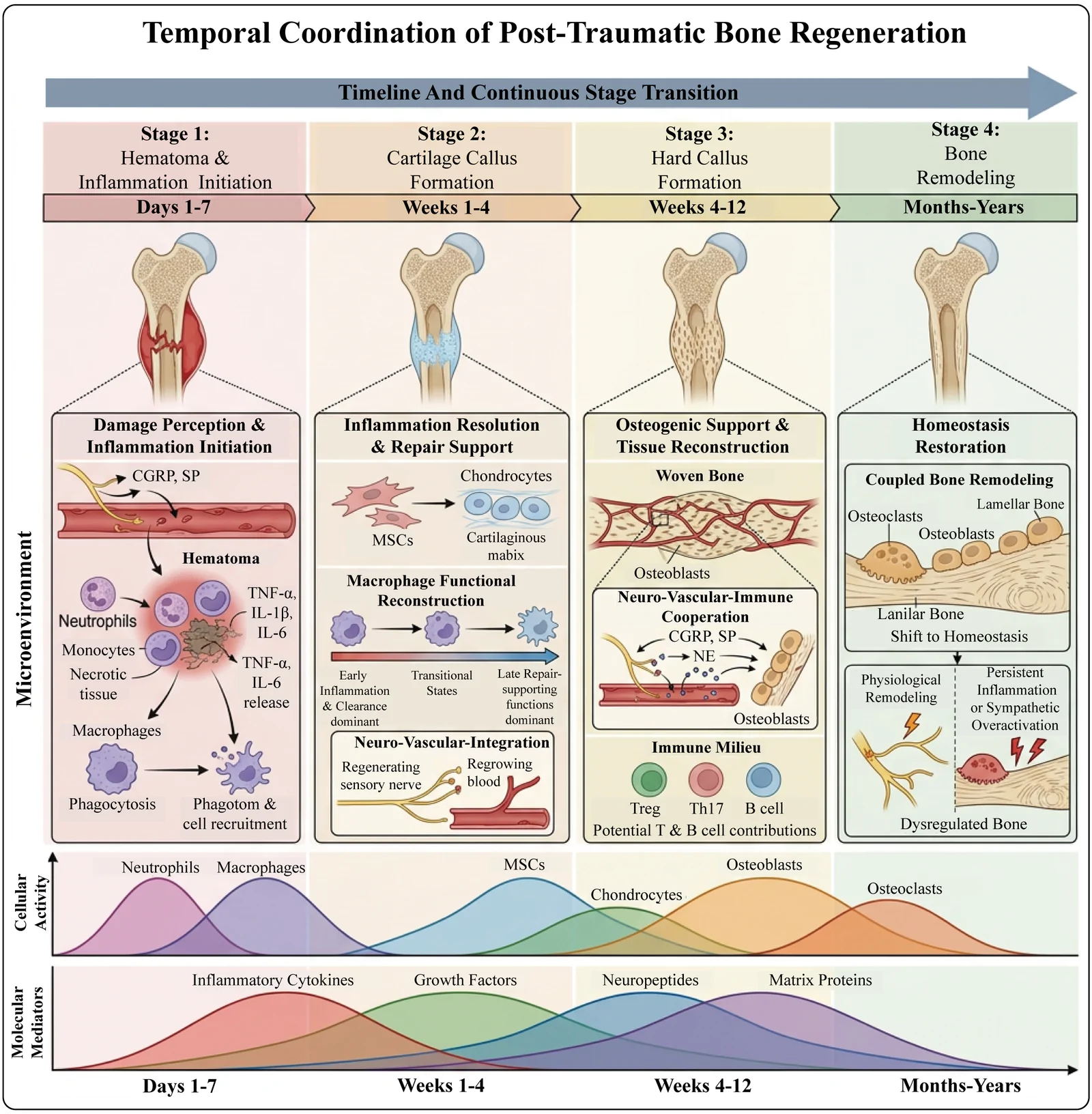
Temporal coordination and continuous stage transitions during post-traumatic bone regeneration. Fracture healing is a dynamic process integrating inflammation, neurovascular reconstruction, endochondral ossification, and bone remodeling. During the early inflammatory phase (days 1–7), hematoma formation, neutrophil and monocyte/macrophage recruitment, and neuropeptide signaling initiate tissue clearance and repair. During cartilage callus formation (weeks 1–4), inflammation progressively resolves, macrophages acquire repair-supportive states, and MSC recruitment, chondrogenesis, and neurovascular regeneration increase. During hard callus formation (weeks 4–12), coordinated neural, vascular, immune, and osteogenic signaling promotes woven bone formation and tissue reconstruction. The subsequent remodeling phase extends from months to years and restores bone homeostasis through coupled osteoblast-osteoclast activity. The lower curves illustrate the relative temporal dynamics of major cellular populations and molecular mediators throughout healing.

**Table 1:** Temporal changes in major immune-cell subsets and functional states across stages of fracture healing.

Repair stage	Primary temporal and histological features	Main functional tendency of macrophages	Treg	Th17	Other major immune cells	Main mediators/ functions	Evidence and limitations
Hematoma/Inflammation Initiation Stage	Early inflammation, tissue clearance	Functions related to inflammation, phagocytosis, debris clearance, and cell recruitment predominate.	Relatively limited; stage-specific role is unclear.	Pro-inflammatory role may be enhanced.	Neutrophils, monocytes	TNF-α, IL-1β, IL-6; damage clearance and inflammation initiation.	Relatively strong evidence for macrophages; limited stage-specific evidence for T cells.
Soft Callus Formation Stage	Gradual resolution of inflammation; recruitment of blood vessels and progenitor cells.	Transitional state increases; functions related to inflammation resolution, pro-angiogenesis, and repair are enhanced.	May contribute to limiting inflammation and maintaining a reparative environment.	Persistent activation may maintain inflammation/osteoclast environment.	Macrophages, T cells	IL-10, TGF-β, VEGF; inflammation resolution, angiogenesis, and tissue repair.	Significant overlap of multiple states; lack of strict stage boundaries.
Hard Callus Formation Stage	Vascular maturation, enhanced osteogenesis, callus mineralization.	Functions related to repair, pro-angiogenesis, and matrix remodeling are prominent.	May support immune homeostasis and repair environment.	Excessive IL-17 signaling may impair bone remodeling.	T cells, B cells, and other lymphoid subsets.	VEGF, TGF-β, RANKL/OPG; osteogenic support and osteoimmunomodulation.	Stage-specific evidence for Treg/Th17 in fracture remains limited.
Remodeling Stage	Osteoblast-osteoclast coupling, restoration of tissue homeostasis.	Functions related to matrix remodeling and homeostasis persist.	May participate in inflammation limitation and bone metabolic homeostasis.	May participate in osteoclast regulation.	T cells, B cells, osteoclast precursors.	RANKL/OPG, IL-10, IL-17; bone remodeling and immune homeostasis.	Insufficient long-term human evidence; relative contributions of different immune subsets are unclear.

## 3 Bone tissue innervation and its dynamic changes after trauma

### 3.1 Distribution characteristics of nerves in bone tissue

Bone tissue functions not only as a mechanical support structure but also possesses a relatively rich innervation. Nerve fibers are distributed throughout the periosteum, bone marrow cavity, peritrabecular regions, and perivascular spaces; notably, the periosteum exhibits the highest innervation density, providing a critical structural basis for pain perception and local post-traumatic regulation (42). Nerve fibers often enter bone along blood vessels and form a relatively dense distribution network around vessels, providing an anatomical basis for local interactions between neural signals and vascular, immune, and bone marrow stromal cells (43, 44).

Currently, sensory nerves and sympathetic nerves are the two types most clearly associated with bone repair. Sensory nerve fibers primarily originate from the dorsal root ganglia; their terminals release neuropeptides, including CGRP and SP, which mediate pain transmission, inflammatory regulation, vascular responses, and bone metabolism (45). Sympathetic nerve fibers mainly release NE and neuropeptide Y (NPY), and influence the functions of bone cells, immune cells, and vascular cells through corresponding receptors (46, 47). Conversely, direct evidence supporting the role of parasympathetic or cholinergic signaling in traumatic bone regeneration remains notably scarce. Existing studies suggest that acetylcholine-related signals may regulate inflammatory responses through the α7 nicotinic acetylcholine receptor (α7nAChR), but whether this constitutes a neural regulatory pathway with clear anatomical and functional significance at the fracture site remains to be verified (48).

### 3.2 Post-traumatic reinnervation and bone repair

Fractures can cause local nerve fiber disruption, degeneration, and altered neuropeptide release, and the repair area typically undergoes varying degrees of reinnervation thereafter. Studies using fracture models have shown that sensory nerve fibers gradually redistribute toward the injured area as callus forms, with their spatial distribution often adjacent to or overlapping with periosteal reactions, neovascularization, and areas of active bone formation (49, 50). This phenomenon suggests that nerve regeneration may be temporally and spatially coordinated with vascularization and callus maturation. However, existing studies report conflicting data regarding nerve fiber density, subtype composition, and reinnervation speed at different repair stages, making it difficult to establish a consistent and definitive temporal sequence of nerve regeneration.

During sensory nerve reinnervation, CGRP and SP are locally relevant signals of particular interest. CGRP can influence VEGF expression and endothelial cell function, and may participate in vascularization, immune regulation, and osteogenic responses (26). SP can regulate vascular permeability and inflammatory cell recruitment through the NK1 receptor, contributing to early injury responses. The local effects of CGRP and SP are related to the repair stage and microenvironment (51, 52). Meanwhile, newly formed blood vessels not only spatially accompany regenerating nerve fibers, but endothelial cells and the perivascular microenvironment may also provide molecular and structural support for neuronal survival, axonal growth, and local reinnervation through VEGF/VEGFR2-NRP1, angiopoietin/Tie receptor signaling (Ang/Tie), and other neurotrophic signals (53). These observations suggest that post-traumatic reinnervation may form a bidirectional coordination process with vascular remodeling, rather than a unidirectional regulation of angiogenesis by nerves.

The dynamic changes of sympathetic nerves may differ from those of sensory nerves. In the early phase of trauma, sympathetic signaling can participate in blood flow regulation, immune responses, and stress modulation; in the later phase of repair, sustained or abnormal NE/β-adrenergic signaling may affect the osteoblast–osteoclast balance and tissue remodeling (54). However, direct evidence from fracture models regarding the regression, regeneration, and spatial redistribution of sympathetic nerve fibers at different post-fracture stages is markedly less than that for sensory nerves. Therefore, assessment of their local effects should integrate nerve fiber distribution, neurotransmitter levels, and repair stage, rather than using systemic sympathetic activity as a proxy for local sympathetic reinnervation at the fracture site.

The strength of evidence from different experimental models also needs to be distinguished. Some studies supporting the involvement of nerves in bone regeneration derive from atypical fracture models such as local denervation, bone defects, and mandibular or alveolar bone injuries (55). These studies show that nerve injury can be accompanied by reduced new bone formation, while supplementation of neural signals such as CGRP can improve bone formation in some models, providing mechanistic evidence for nerve involvement in bone regeneration (56). However, these results cannot be directly extrapolated to physiological reinnervation processes in typical long bone fractures, nor are they sufficient to prove that insufficient nerve regeneration alone can independently cause delayed union or nonunion.

Existing observations suggest that reduced nerve fiber density, delayed reinnervation, or abnormal nerve-vascular coordination may be associated with compromised local repair microenvironments and abnormal healing (57, 58). However, mechanical instability, severe soft tissue injury, ischemia, infection, and persistent inflammation can both impair nerve regeneration and independently affect bone healing (59). Therefore, at the current stage, it is more appropriate to regard reinnervation status as an important component reflecting the local repair process, while its independent causal contribution to delayed union or nonunion still requires further verification through serial temporal observations and nerve-specific functional interventions.

### 3.3 Involvement of peripheral nerves and the autonomic nervous system

Based on current evidence, peripheral sensory nerves and sympathetic nerves have the most direct association with local fracture repair. Sensory nerves modulate local inflammation, vascularization, and bone cell function through signals such as CGRP and SP, while sympathetic nerves primarily govern blood flow, immune status, and the osteoblast-osteoclast balance through NE and β-adrenergic signaling (60). Both types of neural signals exhibit marked stage dependence: transient injury responses may participate in early repair, whereas sustained or imbalanced neural activity may have different effects on subsequent callus formation and remodeling. It should be noted that many mechanistic conclusions are not directly derived from standard traumatic fracture models. For example, local denervation, bone defect, and systemic sympathetic activation models provide important evidence for understanding neural regulation of bone formation, but their injury patterns, local mechanical environments, and degrees of neural disruption do not fully recapitulate those of clinical fractures (61). Therefore, these models are more suitable for supporting potential mechanisms rather than directly proving that the same effects have equivalent magnitude or causal significance in human traumatic fractures.

Regarding parasympathetic/cholinergic signals, existing evidence mainly suggests that α7nAChR-mediated anti-inflammatory effects may indirectly influence bone repair, but direct studies in typical fractures remain limited. Unlike local sensory and sympathetic reinnervation, central and systemic autonomic regulation exerts a more indirect effect on bone repair through the HPA axis, the sympathetic-adrenal-medullary axis, and the resulting stress and neuroendocrine changes, rather than acting directly on the local callus (62). Chronic stress or persistent pain may be accompanied by changes in sympathetic tone and systemic immune-metabolic status, but the clinical association between these factors and fracture healing outcomes currently derives mainly from observational and longitudinal follow-up studies, which are insufficient to prove that they can independently alter the fracture healing process (63) (**Figure 2**).

**Figure 2:**
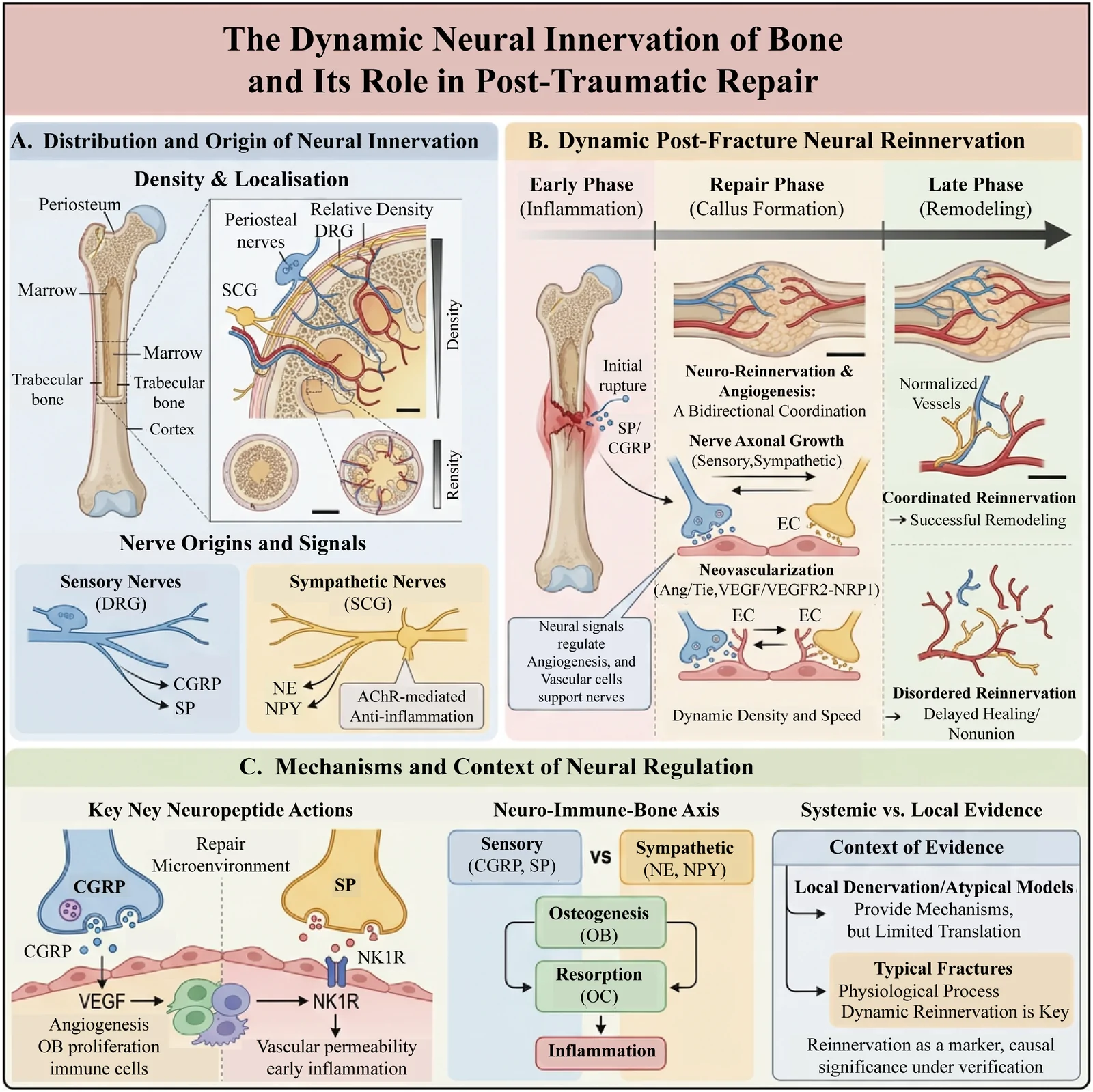
Dynamic innervation of bone tissue and its role in post-traumatic repair. This image outlines the neural distribution in bone tissue, post-fracture reinnervation, and the main mechanisms by which nerves regulate bone repair. **(A)** Distribution and origin of innervation: Nerve density varies across the periosteum, bone marrow, and trabecular bone regions; sensory nerves mainly originate from the DRG and release CGRP and SP, while sympathetic nerves mainly originate from the SCG and release NE and NPY. **(B)** Post-fracture reinnervation dynamics: In the early phase of fracture, nerve disruption occurs with release of signals such as SP/CGRP; during the repair phase, nerve regeneration and angiogenesis occur synergistically to support callus formation; in the later phase, coordinated reinnervation contributes to successful remodeling, whereas disorganized reinnervation may be associated with delayed union or nonunion. **(C)** Mechanisms and evidence background of neural regulation: CGRP can promote angiogenesis, osteoblast proliferation, and immune regulation; SP mainly participates in vascular permeability and early inflammatory responses; sensory and sympathetic signals jointly influence osteogenesis, bone resorption, and inflammation, but the translational significance across different models still requires further validation.

Therefore, the most directly supported evidence at the current stage remains the dynamic reinnervation of sensory and sympathetic nerves in the fracture area and their local interactions with vascular, immune, and bone cells, while systemic neuroendocrine effects should be understood more as an indirect regulatory background. Future research needs to further clarify the spatial distribution, regeneration sequence, and functional contributions of different nerve fibers at each stage of fracture healing through serial time-point tissue imaging, neural subtype labeling, and functional interventions, and to distinguish between correlational and causal relationships among local neural activity, systemic stress changes, and abnormal healing using longitudinal clinical evidence (**Table 2**).

**Table 2:** Evidence levels of bone tissue innervation, post-traumatic dynamics, and their association with bone repair.

Theme	Main nerve/signal Components	Post-traumatic dynamics or main features	Role in bone repair	Evidence level	Main limitations	Reference
Bone Tissue Nerve Distribution	Sensory nerves, sympathetic nerves, and perivascular nerve fibers.	Nerve fibers are distributed in the periosteum, the bone marrow cavity, around trabeculae, and near nutrient vessels, with a higher density of innervation in the periosteum.	Provides anatomical basis for pain perception and local interactions between nerves, blood vessels, immune cells, and bone marrow stromal cells.	Direct anatomical and bone tissue evidence.	Differences in nerve density and subtype distribution may exist across different bone sites, ages, and pathological conditions.	(42–44)
Cholinergic-Related Signals	Acetylcholine, α7nAChR.	May alter local immune responses through the cholinergic anti-inflammatory pathway.	May indirectly support bone repair by limiting inflammation. Evidence of indirect mechanisms.	Evidence of indirect mechanisms.	Direct anatomical and functional evidence in typical traumatic fractures is insufficient to confirm a defined local neural regulatory pathway.	(48)
Sensory Nerves	CGRP, SP, and their related receptors.	Sensory nerve fibers can redistribute to the callus area after fracture, spatially adjacent to periosteal reactions, new blood vessels, and active bone formation areas.	Involved in pain conduction, inflammation regulation, vascular responses, and osteogenic processes.	Direct fracture evidence + bone injury model evidence.	There is a lack of consensus regarding the temporal sequence of nerve fiber density, subtype composition, and reinnervation rate; local effects of CGRP/SP need to be interpreted in conjunction with the stage of repair and microenvironment.	(49–52)
Local Denervation State	Peripheral sensory nerve injury, reduced CGRP, etc.	Some bone injury models show decreased new bone formation after denervation; supplementation with sensory nerve-related signals can improve bone formation in some models.	Supports the possibility that peripheral sensory nerves participate in bone regeneration regulation.	Evidence from other bone injury/denervation models.	The degree of nerve damage and injury pattern in models differ from typical long bone fractures, and cannot directly prove that insufficient nerve regeneration independently leads to nonunion.	(55, 56)
Nerve Reinnervation – Neurovascular Coupling	Sensory nerve fibers, vascular endothelial cells, VEGF/VEGFR2–NRP1, Ang/Tie, etc.	Regenerating nerve fibers are often spatially adjacent to new blood vessels; the vascular and perivascular environment may reciprocally support nerve survival and axon growth.	May coordinate nerve reinnervation, vascular remodeling, and callus maturation.	Fracture observation evidence + other neurovascular/regeneration model evidence.	Direct temporal and functional causal evidence for “vessel → nerve” in fractures remains limited.	(57, 58)
Sympathetic Nerves	NE, NPY, β-adrenergic receptor-related signals.	May be involved in blood flow, immune responses, and bone remodeling after trauma; temporal data on local sympathetic reinnervation are relatively limited.	Can influence immune status, osteoblast-osteoclast balance, and local blood flow.	Limited fracture-related evidence; more from bone homeostasis, stress, and systemic sympathetic models.	Local sympathetic reinnervation cannot be equated with systemic sympathetic activity, and its independent contribution at different fracture stages remains unclear.	(60, 61)
Systemic Neuroendocrine Regulation	HPA axis, SAM axis, glucocorticoids, catecholamines.	Trauma, chronic stress, and chronic pain may be accompanied by changes in systemic neuroendocrine and immune-metabolic environments.	May indirectly affect fracture recovery through systemic stress, immune, and metabolic alterations.	Systemic mechanism evidence + observational/longitudinal clinical evidence.	It is difficult to distinguish systemic neuroendocrine effects from local neural activity in fractures; current evidence is insufficient to prove that these factors can independently alter the fracture healing process.	(62, 63)

## 4 Mechanisms of neuro-immune interaction in post-traumatic bone regeneration

### 4.1 Mechanistic framework of bidirectional neuro-immune interaction

Following trauma, local nerve injury, neuropeptide release, and immune cell recruitment overlap temporally, establishing a close connection between neural activity and inflammatory responses (64, 65). SP and CGRP derived from sensory nerves, as well as NE derived from sympathetic nerves, can act on macrophages, neutrophils, T cells, and vascular cells; conversely, inflammatory cytokines, chemokines, and neurotrophic factors released by immune cells can also regulate nerve regeneration and sensory sensitization, forming bidirectional neuro-immune communication (66).

Although different neurotransmitters have distinct receptors and proximal signaling pathways, their immune effects can further converge on shared intracellular regulatory nodes such as NF-κB, Mitogen Activated Protein Kinases (MAPK), cAMP-response element binding protein (CREB), and JAK/STAT. These signals translate neural input into distinct immune responses by orchestrating inflammation-related gene transcription, cytokine production, and immune cell functional states (67). Therefore, rather than viewing CGRP, SP, or NE as mutually independent regulatory factors, it is more appropriate to understand neuro-immune communication from the hierarchical perspective of “receptor-intracellular signaling-transcriptional network-cellular state”.

Notably, the strength of evidence supporting this mechanistic network varies. While some observations are derived directly from traumatic fracture models, others stem from bone defect, denervation, or craniomaxillofacial injury models; meanwhile, specific receptor-signaling and transcriptional mechanisms rely primarily on data from general neuroimmunology, peripheral nerve injury, and pain/inflammation studies (68). Thus, signaling nodes such as NF-κB, MAPK, CREB, and JAK/STAT serve as important mechanistic frameworks for understanding how different neuro-immune inputs converge. However, their specific contributions at different stages and in different cell types during traumatic fracture still require evaluation based on experimental models and endpoint evidence, highlighting that general neuro-immune mechanisms cannot be directly equated with complete causal pathways in fracture healing.

### 4.2 Regulation of the immune microenvironment by sensory neuropeptides

CGRP and SP are currently the most extensively studied sensory neuropeptides, but their effects cannot be rigidly classified as “anti-inflammatory” or “pro-inflammatory,” given their dependence on receptor expression, local concentration, duration of action, and target cell state. CGRP primarily acts through the calcitonin receptor-like receptor/receptor activity-modifying protein 1(CALCRL/RAMP1) receptor complex (69). In fracture and certain bone injury models, more direct evidence primarily supports its association with angiogenesis, osteogenic responses, and callus repair (70). In immune cells, CGRP binding to CALCRL/RAMP1 activates cAMP-PKA signaling and influences inflammatory gene expression through CREB and its cross-regulation with NF-κB-related transcriptional programs (71). CREB activation is associated with immune regulation and cell survival, while NF-κB serves as a critical transcriptional regulatory node for multiple pro-inflammatory genes including TNF-α, IL-1β, and IL-6 (72). Therefore, the effect of CGRP on immune status is more likely reflected in the coordination of CREB and NF-κB transcriptional networks rather than a single “anti-inflammatory” effect (73, 74). In contrast, evidence for CGRP promoting Treg expansion or inducing specific macrophage repair programs is drawn largely from general immunology and other tissue injury models, and its direct causal contribution in the context of traumatic fracture still requires further validation (75).

SP primarily mediates neurogenic inflammation and immune regulation through NK1R. In the early phase of injury, SP release can be accompanied by increased vascular permeability and inflammatory cell recruitment (65, 76). Upon neurokinin 1 receptor (NK1R) activation, phospholipase C (PLC) subsequently activates MAPK branches including ERK, p38, and JNK, and promotes NF-κB activation, thereby regulating the expression of inflammation-related genes such as TNF-α, IL-1β, IL-6, and chemokines (77). MAPK and NF-κB thus constitute important nodes that convert SP-mediated neurogenic input into inflammatory transcriptional responses. These receptor–intracellular signaling relationships have relatively clear experimental support, but the full pathological cascade linking sustained SP signaling to chronic inflammation and aberrant bone repair relies primarily on indirect evidence from inflammation and pain models. Therefore, the role of SP in fracture repair is better understood as a stage and microenvironment-dependent regulatory signal rather than a sustained pro-inflammatory factor.

From a signal transduction perspective, CGRP and SP represent distinct yet overlapping sensory neuro-immune regulatory patterns: CGRP primarily operates through CALCRL/RAMP1-cAMP/PKA-CREB and modulates NF-κB-related transcriptional programs, while SP primarily connects neurogenic stimuli to inflammatory gene expression through NK1R-PLC-MAPK/NF-κB (69, 78). NF-κB and MAPK thus emerge as important downstream nodes where different sensory neural inputs converge. Other neuropeptides such as NPY, VIP, and PACAP can also affect macrophage, T cell, and B cell functions, but direct studies in typical traumatic fractures are limited, and they currently provide supplementary mechanistic clues.

### 4.3 Sympathetic neuro-immune regulation

Unlike sensory nerves, sympathetic nerves primarily influence bone cells and immune cells through NE and adrenergic receptors, among which β-2 adrenergic receptor (β2-AR) is the most extensively studied receptor subtype (79). Sustained β2-AR signaling is associated with suppressed osteogenesis, altered RANKL/OPG balance, and enhanced bone resorption (80). However, a considerable portion of these conclusions derives from studies on bone homeostasis, systemic sympathetic activation, or chronic stress models, and therefore primarily supports the mechanistic possibility that sympathetic nerves participate in bone metabolic regulation, but is insufficient to prove that sustained sympathetic activation can independently cause impaired fracture healing.

In immune cells, NE can alter the functions and cytokine release profiles of macrophages, neutrophils, and T cells (81). As a Gs-coupled receptor, β2-AR activates cAMP-PKA signaling and influences inflammatory gene expression and cytokine production through CREB and its cross-regulation with NF-κB-related transcriptional programs (82). CREB is more involved in cAMP-dependent transcriptional regulation, while NF-κB serves as an important integrative node for multiple pro-inflammatory gene expressions; thus, the two may jointly determine the specific nature of the immune response resulting from sympathetic input (83). Some experiments suggest that transient or moderate β2-AR activation can limit excessive inflammation, whereas sustained or aberrant sympathetic input may interfere with inflammation resolution and tissue repair (84). This difference indicates that β2-AR signaling does not possess a fixed “pro-inflammatory” or “anti-inflammatory” effect; rather, its outcome depends on signal intensity, duration, and the local cytokine environment in which immune cells reside.

The STAT family is better understood as secondary transcriptional integration nodes following sympathetic signaling-induced changes in the cytokine environment, rather than direct downstream effectors of NE-β2-AR (85). For example, IFN-γ-JAK/STAT1 is typically associated with inflammation-related transcriptional programs, IL-10-JAK/STAT3 mediates the resolution of inflammation and immune regulation, and IL-4/IL-13-JAK/STAT6 is more associated with tissue repair-related programs (86). Therefore, sympathetic regulation of immune status can be understood as a hierarchical process: β2-AR-cAMP/PKA first influences initial transcriptional responses and cytokine release through CREB/NF-κB, and the subsequently altered cytokine environment further shapes immune cell functional states through STAT1, STAT3, or STAT6. This framework also avoids simplifying NE action to directly inducing a fixed macrophage phenotype.

Under chronic stress and persistent pain conditions, the aforementioned regulation may undergo sustained deviation. Long-term activation of the HPA axis and the sympathetic-adrenal-medullary axis can alter glucocorticoid and catecholamine levels, and reshape the bone repair microenvironment by affecting immune cell function, osteogenesis, and bone resorption (87). Persistent pain may also be accompanied by enhanced sympathetic tone and sensory nerve sensitization, leading to long-term imbalance of neural signals such as NE, SP, and CGRP (88). However, systemic sympathetic activation and local sympathetic reinnervation in fracture are phenomena at different levels, and there is currently a lack of temporal studies comparing β2-AR-CREB/NF-κB and STAT networks continuously across different fracture stages. Therefore, existing evidence suggests that sympathetic activity may alter the local immune and bone metabolic milieu in fracture through multi-level transcriptional networks, but its independent contribution to final callus formation and healing outcomes still requires fracture-specific functional studies and longitudinal clinical evidence for validation.

### 4.4 Reverse regulation of nerve regeneration and sensory sensitization by immune cells

Neuro-immune communication is not solely directed from the nervous system to the immune system; immune cells can also exert retrograde regulation on neural function through neurotrophic factors, inflammatory mediators, and metabolic signals. Macrophages can release NGF, BDNF, GDNF, as well as CCL2, CXCL1, and other molecules, thereby affecting axonal growth, neuronal excitability, and tissue reinnervation (89, 90). In peripheral nerve injury and other tissue regeneration models, macrophages with tissue-repair characteristics are more frequently associated with neurotrophic factor production and axonal extension, whereas macrophages in persistent inflammatory states can enhance neuroinflammation and sensory sensitization through mediators such as TNF-α, IL-1β, and ROS (91). These results support that different macrophage functional states may produce distinct neural effects through neurotrophic and inflammatory signals, but whether the same mechanisms can directly determine the quality of nerve reinnervation in fracture callus remains insufficiently evidenced.

T cells may also participate in this feedback process. Treg-derived IL-10 and TGF-β are associated with the resolution of inflammation and tissue repair, whereas Th17/IL-17 signaling is primarily associated with inflammation amplification (92). At the downstream signaling level, IL-10 participates in immune regulation through JAK/STAT3, TGF-β primarily engages SMAD2/3 transcriptional programs, and IL-17 can activate NF-κB and MAPK-related inflammatory signals (93). These pathways have sufficient general immunological evidence, but whether Treg or Th17 directly controls nerve fiber regeneration in the fracture area through the aforementioned signals has not been specifically validated; their effects are more likely first reflected in reshaping the local inflammatory environment and thereby indirectly affecting neural recovery.

In contrast, the molecular mechanisms by which inflammatory mediators induce sensory nerve sensitization are more clearly established in neuroinflammation and pain research. TNF-α/TNFR can connect NF-κB and MAPK signaling, IL-1β/IL-1R primarily activates NF-κB and p38 MAPK, and IL-6 can regulate neuronal inflammatory responses and excitability through JAK/STAT3 (94). These downstream nodes can convert cytokine input into inflammation-related gene expression, cellular stress, and changes in neuronal excitability, thereby connecting the immune microenvironment with sensory nerve function. After sensory nerve sensitization, the release of neuropeptides such as SP and CGRP can increase, further acting on immune cells and blood vessels, forming a feedback loop of “immune mediators-neural sensitization-neuropeptide release” (95, 96). This feedback has substantial evidence in pain and neuroinflammation models, but its duration during normal fracture repair and whether it further affects callus formation and final healing outcomes still lack direct validation.

Persistent inflammation can also alter neural function through ROS, proteases, and neurotransmitter metabolism, further linking immune status with neural recovery (97, 98). From the perspective of the entire neuro-immune network, NF-κB, MAPK, and JAK/STAT are not exclusively specific downstream effectors of any single neurotransmitter or cytokine, but rather constitute shared signal integration nodes among neural signals, inflammatory mediators, and immune cell states (99). Among these, NF-κB and MAPK primarily participate in relatively rapid inflammatory and stress transcriptional responses, while the STAT family more integrates sustained cytokine environments and participates in the subsequent shaping and maintenance of immune functional states (100). The resulting cycle of “neural regulation of immunity-immune feedback to nerves” provides a mechanistic framework for understanding the interactions among local inflammation, nerve reinnervation, and pain after trauma, although the robustness of the evidence varies across different components (**Figure 3**).

**Figure 3:**
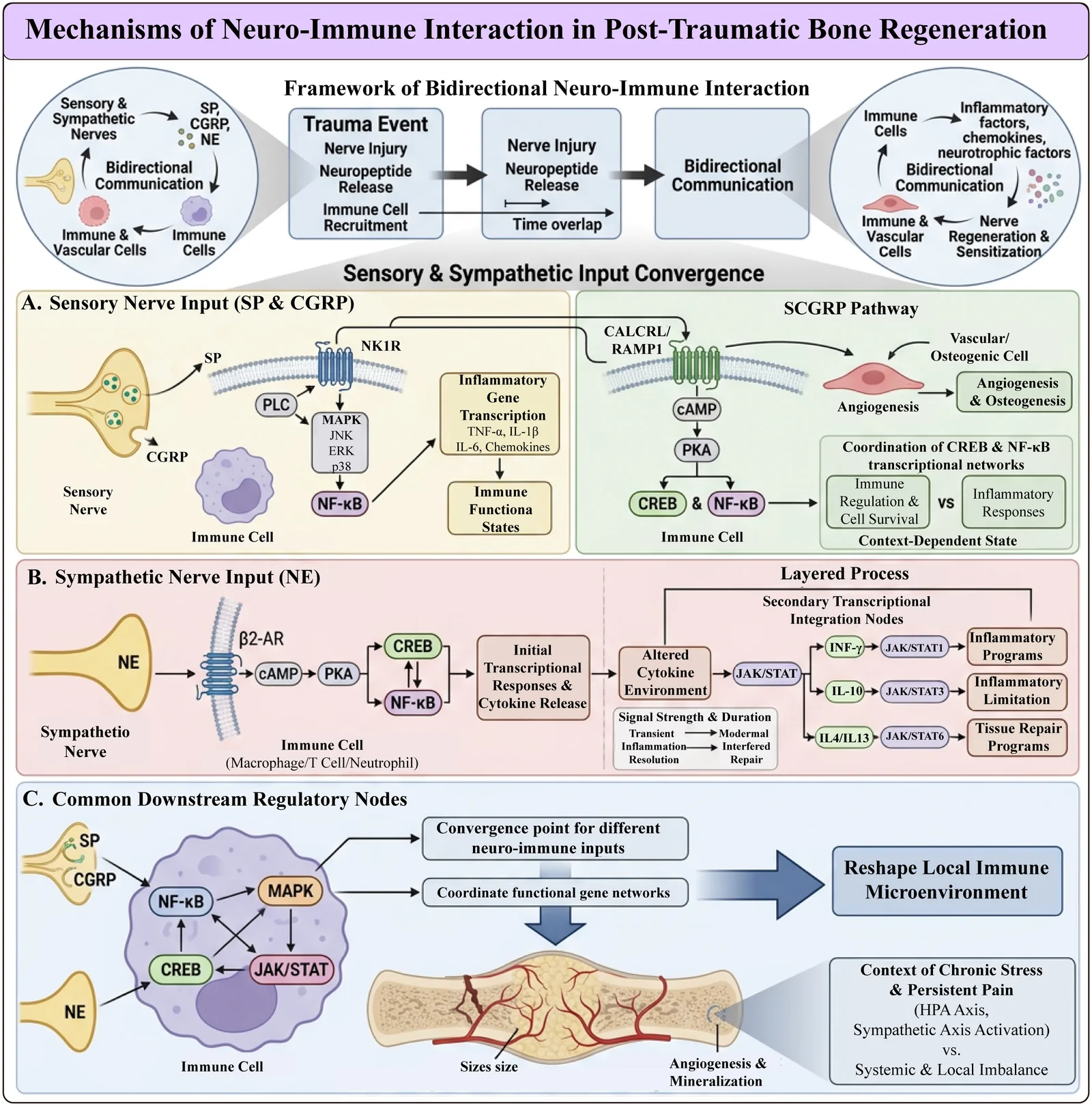
Neuro-immune interaction mechanisms in post-traumatic bone regeneration. This image outlines the bidirectional communication between sensory nerves, sympathetic nerves, and immune cells after fracture and its regulatory effects on the bone repair microenvironment. **(A)** Sensory neural input: Following injury, sensory nerves release SP and CGRP; SP can induce inflammatory gene expression through the NK1R-PLC-MAPK-NF-κB pathway, while CGRP can regulate CREB and NF-κB networks through CALCRL/RAMP1-cAMP-PKA signaling, thereby modulating immune cell states, angiogenesis, and osteogenesis. **(B)** Sympathetic neural input: NE acts on macrophages, T cells, and neutrophils through the β2-AR-cAMP-PKA pathway, regulating early cytokine release and influencing inflammation resolution and tissue repair through JAK/STAT-related secondary transcriptional networks. **(C)** Shared downstream regulatory nodes: SP, CGRP, and NE signals converge at nodes including NF-κB, MAPK, CREB, and JAK/STAT, reshaping the local immune microenvironment and further affecting angiogenesis, mineralization, and callus repair; chronic stress and persistent pain may disrupt systemic and local neuro-immune homeostasis.

Therefore, neuro-immune interaction is better understood as a bidirectional regulatory system in which multiple receptors and intracellular signaling networks converge. At the molecular level, receptor–downstream signaling relationships including SP-NK1R-MAPK/NF-κB, CGRP-CALCRL/RAMP1-cAMP/PKA/CREB, NE-β2-AR-cAMP/PKA, and IL-6/IL-10-JAK/STAT3 are supported by robust experimental evidence; in contrast, the complete causal chain by which these molecular events further drive specific macrophage states, T cell responses, local nerve reinnervation in fracture, and ultimately healing outcomes remains insufficiently validated. Therefore, the existing mechanistic network should be interpreted based on the origin and robustness of the supporting evidence, encompassing both established molecular connections and candidate pathways supported by other injury models as well as those still requiring validation in traumatic fracture models (**Table 3**).

**Table 3:** Major neuro-immune interactions and their evidence levels in post-traumatic bone regeneration.

Neural mediator	Immune/cellular target	Receptor/main pathway	Main bone repair-related effect	Evidence source	Suggested evidence level
CGRP	Macrophages, dendritic cells, T cells	CALCRL/RAMP1-cAMP/PKA-CREB.	Inflammatory regulation, angiogenesis, osteogenic support	Fracture/bone defect + general immune studies.	Moderate/ indirect
CGRP	Treg	CALCRL/RAMP1 and downstream immunomodulation.	Potential induction of acquired immune tolerance and limitation of inflammation.	Primarily non-fracture immune models.	Indirect
SP	Macrophages, neutrophils, mast cells	NK1R-PLC/MAPK/NF-κB.	Recruitment of inflammatory cells and mediation of neurogenic inflammation.	Injury models + general inflammation studies.	Moderate/indirect
NE	Macrophages, T cells	β2-AR–cAMP/PKA-CREB/NF-κB.	Alteration of cytokine profiles and modulation of the bone immune environment.	Primarily systemic/bone metabolism and immune models.	Indirect
Macrophage cytokines	Sensory nerves/axons	NGF/BDNF/GDNF, etc.	Axonal growth, reinnervation.	Primarily peripheral nerve injury models.	Indirect
TNF-α/IL-1β/IL-6	Sensory nerves	NF-κB/MAPK/JAK-STAT.	Neuronal sensitization, pain amplification.	Pain/inflammation models.	Mechanistic evidence is strong, though primarily extrapolated from fracture models.
Treg/Th17 cytokines	Nerves/macrophages	STAT3, SMAD2/3, NF-κB/MAPK.	Potential limitation or amplification of the inflammatory response	Primarily non-fracture models.	Limited/ candidate

## 5 Effects of the neuro-immune-bone axis on bone formation, angiogenesis, and bone remodeling

### 5.1 Effects on bone marrow mesenchymal stem cell recruitment and osteogenic Fate

Bone marrow mesenchymal stem cells (BMSCs) are a critical source of chondrocytes and osteoblasts during fracture repair, and their recruitment, survival, and differentiation are co-regulated by neural signals, immune status, vascularization, and the local metabolic environment (101, 102). Rather than viewing a single neurotransmitter as an independent determinant of BMSC fate, a more reasonable understanding is that neural input can act directly on BMSCs or indirectly modulate their osteogenic potential by altering the immune and vascular microenvironment.

To date, the most well-characterized direct neural effects have primarily been derived from studies focusing on CGRP. Studies utilizing various *in vitro* cell cultures, bone defect, and bone repair models demonstrate that BMSCs express CGRP-associated receptors, and that CGRP stimulation enhances osteogenic gene expression and mineralization capacity (103, 104). These results support the possibility that sensory nerve signals directly regulate the osteogenic program of mesenchymal cells, but the cell sources, injury models, and CGRP exposure methods used in different studies are inconsistent, making it difficult to quantify their relative contribution to BMSC differentiation in typical fractures. Conversely, the effects of SP and NE on BMSCs appear highly context-dependent, with their actions potentially mediated either by direct cell receptor signaling or indirectly via the immune-inflammatory milieu (105–107). Therefore, the final osteogenic outcome of BMSCs cannot be inferred solely from changes in neurotransmitter levels.

The immune microenvironment may serve as an important mediator in this regulation. Macrophages can produce TGF-β, VEGF, and other repair-related factors to support BMSC recruitment, survival, and differentiation; persistent inflammatory signals such as TNF-α, IL-1β, and IL-6 may inhibit the osteogenic program and increase cellular stress (108–110). Treg-related immunomodulatory signals may also provide a more favorable repair environment for BMSCs by limiting persistent inflammation, but direct evidence in traumatic fractures remains limited. Thus, the effect of neuro-immune interactions on BMSCs is more likely to be a combined result of direct neural input and immune/vascular microenvironment-mediated effects. Current experimental studies can support some direct neural-BMSC connections but are insufficient to determine the relative weight of direct neural signals versus immune-mediated effects in fracture healing *in vivo*.

### 5.2 Effects on osteoblast-osteoclast coupling

Fracture repair does not simply involve increasing bone formation but relies on the dynamic coupling of osteoblasts and osteoclasts during callus formation, mineralization, and remodeling. Neuro-immune interactions can influence this process at two levels: on one hand, neural signals can directly act on osteoblasts or osteoclasts and their precursors (111); on the other hand, neural signals can first alter the local immune state, then indirectly change the osteoblast-osteoclast balance through RANKL/OPG, inflammatory cytokines, and other osteoimmune signals (112).

Experimental studies have suggested that CGRP can enhance osteoblast differentiation and bone matrix formation and, in some systems, inhibit RANKL-induced osteoclastogenesis (75). NE/β-adrenergic signaling is associated with osteoblast inhibition, altered RANKL/OPG balance, and enhanced bone resorption (113). However, a significant portion of these direct cellular effects is derived from *in vitro* cultures, bone homeostasis models, or systemic neural regulation models and may not fully reflect the actual contribution of local neural signals in traumatic fractures. The effects of SP on osteoblasts and osteoclasts are also model-dependent, and its net contribution to osteoblast-osteoclast coupling in traumatic fractures remains unclear.

Immune cells provide an important intermediate layer connecting neural input to bone cell responses. Macrophages, T cells, and B cells can regulate osteoclastogenesis through signals such as RANKL, TNF-α, IL-1β, IL-6, and IL-17, while signals like IL-10 and TGF-β mitigate persistent inflammation and excessive bone resorption (111, 114). Studies from inflammatory bone destruction models further indicate that the RANKL/RANK/NFATc1 signaling pathway is crucial for driving osteoclastogenesis and bone resorption, but such evidence mainly reflects pathological bone destruction, and its specific contribution to traumatic fracture remodeling needs to be assessed in fracture models (115). Therefore, when neural activity first alters immune cell function, the ultimate impact on bone formation or resorption is likely mediated indirectly through the osteoimmune network rather than directly acting on osteoblasts or osteoclasts.

Osteoblast-osteoclast coupling also involves intercellular communication networks such as Wnt/Sclerostin, RANKL/OPG, and Ephrin/Eph (116, 117). Persistent sympathetic activation or chronic inflammation may alter these coupling signals, biasing callus remodeling toward bone resorption, but there is currently insufficient evidence to demonstrate that neuro-immune signals can dictate the osteoblast-osteoclast transition in fractures through a single pathway. Therefore, the neuro-immune system is more likely to influence bone remodeling by modulating the local environment of osteoblast-osteoclast coupling rather than unidirectionally controlling a specific bone cell type. Future research particularly needs to distinguish the relative contributions of direct neural-bone cell actions versus immune cell-mediated indirect effects at different repair stages.

### 5.3 Effects on endothelial cells, angiogenesis, and neurovascular reconstruction

New blood vessels provide oxygen and nutrients to the fracture area and constitute an important tissue interface for the migration and homing of immune cells, BMSCs, and osteogenic precursor cells (118). Therefore, in the process of neuro-immune regulation of bone regeneration, vascular endothelial cells are not merely passive blood supply carriers but also important effector cells connecting nerve reinnervation, immune status, and osteogenic responses. Some experimental studies suggest that sensory nerve signals can directly act on endothelial cells. CGRP is associated with endothelial cell proliferation, VEGF-related signaling, and angiogenesis, while SP can regulate vascular permeability and endothelial cell migration (119, 120). Sympathetic nerve signals can also alter endothelial cell function, but their effects are influenced by receptor subtypes, signal intensity, and tissue environment (121). Furthermore, immune cells can shape the angiogenic microenvironment; for example, macrophage-derived VEGF, platelet-derived growth factor (PDGF), and fibroblast growth factor 2 (FGF-2) can promote vascular sprouting and maturation, while persistent inflammation may impair endothelial barrier function and microvascular stability (122). Thus, the ultimate effect of neural signals on angiogenesis may result from direct “neural-endothelial” actions or be indirectly mediated by immune cells and the local inflammatory environment.

Neurovascular coupling also exhibits a bidirectional regulatory capacity, where new blood vessels and their surrounding microenvironment can influence nerve survival and reinnervation. In addition to its classic pro-angiogenic effects, VEGF can affect neuronal survival, axon extension, and growth cone guidance through VEGFR2 and neuropilin-1 (NRP1)-related signaling, providing a mechanistic basis for shared molecular cues between angiogenesis and nerve regeneration (123). In contrast, the Ang1/Ang2-Tie2 axis currently has a more defined role in regulating vascular maturation, stability, and remodeling. Its impact on nerve regeneration is likely indirect, mediated by maintaining perivascular structures, improving local perfusion, and remodeling the microenvironment essential for nerve growth; currently, insufficient evidence exists to demonstrate that it directly promotes axonal regeneration in traumatic fracture areas (124). Additionally, glial cell line-derived neurotrophic factor (GDNF) family factors such as Artemin can support sensory nerve axon growth and guidance through corresponding neurotrophic receptors, providing another potential mechanism for the perivascular environment to promote nerve reinnervation.

These findings suggest that angiogenesis and nerve reinnervation after fracture may not be two independent processes but rather form a bidirectional coupling through shared molecular signals and spatial microenvironments: neural signals can regulate endothelial and immune cells, while blood vessels and their surrounding environment can provide nutrients, structural scaffolds, and neurotrophic cues for regenerating nerves. However, current direct mechanistic evidence for “endothelial/vascular → nerve” effects primarily comes from neurovascular biology, peripheral nerve injury, and other tissue regeneration models, and the spatiotemporal relationships and functional contributions in typical traumatic fractures lack sufficient validation. Therefore, existing evidence supports a synergistic relationship between nerve reinnervation and vascular reconstruction but is insufficient to regard single signals like VEGF or Ang/Tie as independent causal mechanisms determining nerve regeneration in fracture areas.

### 5.4 “Neuro-vascular-immune-bone regeneration niche”: an integrative conceptual model

The above studies suggest that nerve fibers, vascular endothelial cells, immune cells, BMSCs, and bone cells in the callus are not independent but form multi-level connections through neuropeptides, cytokines, growth factors, and intercellular communication within a shared spatial environment (125, 126). Based on these disparate yet interconnected lines of evidence, this paper proposes the “Neuro-Vascular-Immune-Bone Regeneration Niche” as an integrative conceptual model for understanding post-traumatic multi-cellular coordinated repair. It should be clarified that this “niche” is not currently an independent anatomical or biological structure confirmed by spatial omics, lineage tracing, or multi-cellular functional interventions but rather a working framework for integrating existing evidence on neural, vascular, immune, and bone repair. Its core hypothesis is that in the fracture repair area, nerve reinnervation, vascular reconstruction, immune state transition, and osteoprogenitor cell differentiation may be coordinated within specific spatial neighborhoods, allowing different cell populations to share and exchange repair signals.

Within this framework, nerve fibers can deliver local signaling molecules such as CGRP, SP, and NE; immune cells participate in inflammation resolution and growth factor environment regulation; blood vessels provide perfusion, cell migration channels, and a perivascular environment supporting nerve growth; and BMSCs, osteoblasts, and osteoclasts collectively orchestrate callus formation and remodeling programs (127). Among these, VEGF-related neurotrophic signals and Ang/Tie-mediated vascular stabilization and perivascular environment remodeling may constitute important molecular and structural bases for vascular reconstruction supporting nerve reinnervation (128). However, while experimental support exists for some of these intercellular connections, direct evidence remains lacking regarding their ability to form a stable, identifiable, and functionally independent spatial niche within the fracture callus.

This concept also provides a new perspective for understanding abnormal healing. Ischemia, persistent inflammation, abnormal nerve reinnervation, or impaired osteoprogenitor cell function may simultaneously disrupt multiple repair processes, leading to temporal or spatial desynchronization (129). However, current evidence is insufficient to identify “niche disruption” itself as a direct cause of delayed union or nonunion; a more reasonable explanation is that multi-system mismatch may be a common feature of the abnormal bone repair microenvironment. Future research needs to combine spatial transcriptomics, multiplex tissue imaging, neural and vascular subtype markers, single-cell analysis, and temporal functional interventions to simultaneously localize nerve fibers, immune cells, vascular endothelial cells, and osteoprogenitor cells in the same fracture model and further verify whether the formation, disruption, or restoration of specific spatial neighborhoods can alter bone healing outcomes. Only after obtaining clear spatial and functional evidence can the “Neuro-Vascular-Immune-Bone Regeneration Niche” evolve from an integrative conceptual model into a repair unit with defined biological boundaries and functional significance (**Figure 4**).

**Figure 4:**
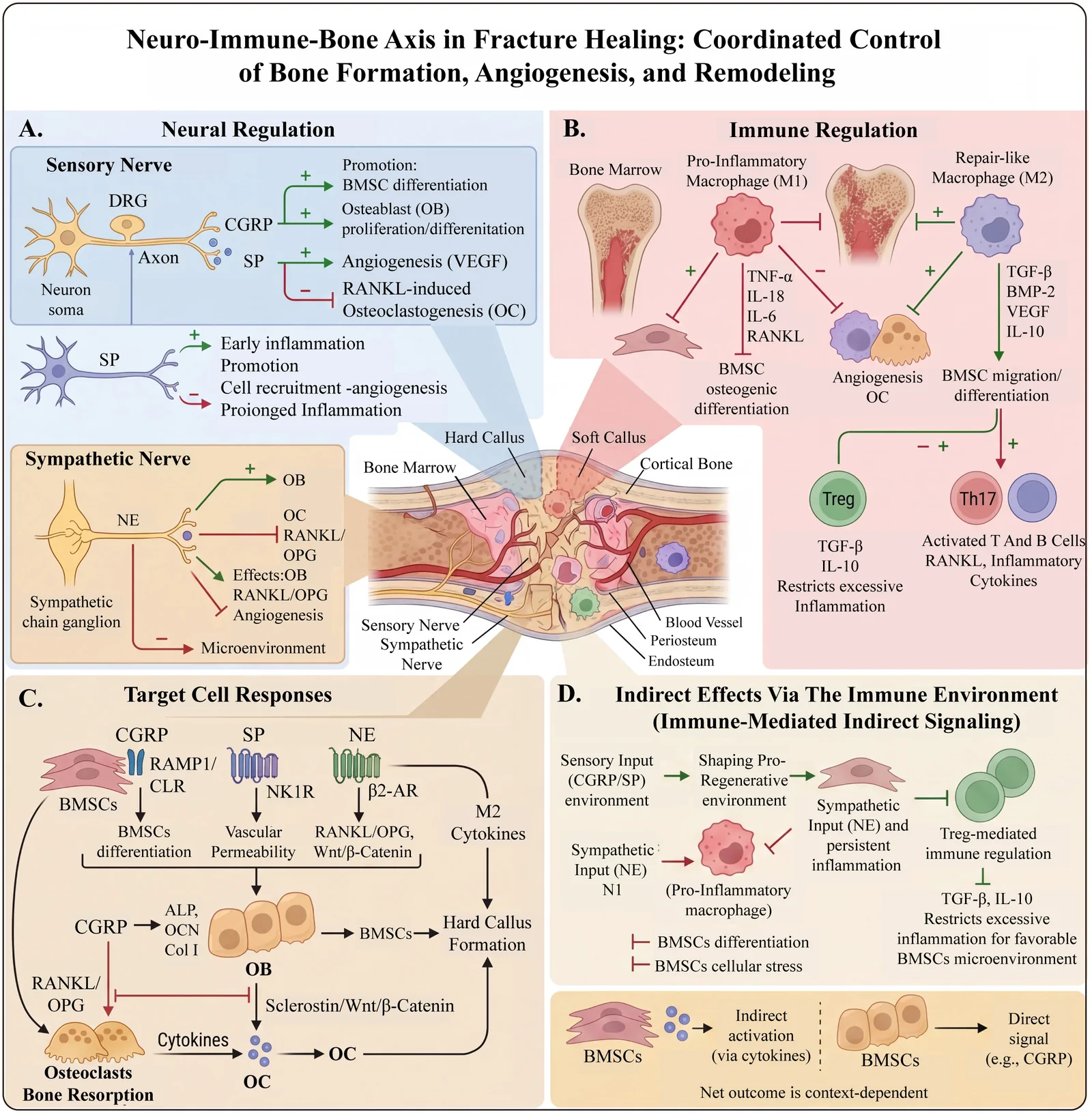
Synergistic regulation of osteogenesis, angiogenesis, and remodeling by the neuro-immune-bone axis in fracture healing. This image outlines the interactions of sensory nerves, sympathetic nerves, immune cells, and bone lineage cells during fracture repair. **(A)** Neural regulation: Sensory nerves release CGRP and SP, promoting BMSC differentiation, osteoblast proliferation, and VEGF-related angiogenesis, while influencing osteoclast activity; sympathetic nerve-derived NE regulates osteogenesis, osteoclastogenesis, and the local microenvironment through pathways such as β2-AR. **(B)** Immune regulation: M1-like macrophages and inflammatory cytokines mediate the early inflammatory response, while M2-like macrophages, Tregs, and repair-related factors contribute to the resolution of inflammation, angiogenesis, and BMSC migration/differentiation. **(C)** Target cell response: BMSCs, osteoblasts, and osteoclasts integrate CGRP, SP, and NE signals to regulate bone formation, bone resorption, and hard callus formation. **(D)** Indirect effects mediated by the immune environment: Neural input can indirectly influence BMSC function and bone regeneration by shaping the local immune status, with the ultimate effect depending on the degree of inflammation, sympathetic activation, and repair stage.

## 6 Neuro-immune-bone axis imbalance and impaired bone healing

### 6.1 Delayed healing and nonunion: imbalance phenotypes and causal boundaries

Delayed healing and nonunion result from multiple interacting factors, influenced by mechanical stability, local blood supply, infection, bone defect extent, soft tissue injury, and host metabolic status (130). Neuro-immune-bone axis abnormalities are more appropriately viewed as one potential pathological regulatory layer rather than an independent or sole pathogenic mechanism. Normal bone repair requires relative temporal and spatial coordination among reinnervation, inflammation resolution, angiogenesis, and osteoblast-osteoclast coupling, whereas delayed healing or nonunion regions often exhibit varying degrees of discordance among these processes (131, 132).

Some animal experiments and clinical observations have shown that regions exhibiting abnormal healing may exhibit reduced sensory nerve fiber density and altered CGRP and SP expression (5, 133). However, these changes may reflect insufficient reinnervation or may be secondary to severe soft tissue injury, ischemia, persistent inflammation, or prolonged mechanical instability; thus, a unidirectional causal relationship of “neuropeptide reduction leading to nonunion” cannot yet be established. In abnormal healing, altered CGRP or SP levels must be interpreted in the context of the repair stage at which they occur, and mere elevation or reduction in expression does not determine their pathological significance. Therefore, whether neuropeptide signaling matches the repair stage is more worthy of attention than absolute changes in levels.

Abnormal healing is also frequently accompanied by persistent inflammation and impaired immune status transition. Sustained elevations of inflammatory mediators such as TNF-α, IL-1β, and IL-6 can suppress BMSC and osteoblast function and enhance osteoclastic responses through RANKL-related pathways. Meanwhile, persistent inflammation may also affect vascular stability and neural recovery (134). However, chronic inflammation may either contribute to healing impairment or be a consequence of infection, tissue necrosis, mechanical instability, or prolonged nonunion (135). Therefore, “persistent inflammation-limited neural recovery-impaired osteogenesis” is more appropriately regarded as an interrelated pathological network in abnormal healing rather than an established linear causal chain.

Sympathetic nervous system abnormalities may also participate in this process. Persistent pain, chronic stress, and metabolic abnormalities can be accompanied by altered NE/β-adrenergic signaling, further affecting immune cells, osteoblasts, and osteoclasts (136–138). However, systemic sympathetic hyperactivity, abnormal local sympathetic reinnervation, and fracture healing impairment operate at different biological levels, and current evidence is insufficient to demonstrate that sympathetic dysregulation can independently cause delayed healing or nonunion (80, 136). Therefore, abnormal reinnervation, persistent inflammation, and altered sympathetic signaling in delayed healing and nonunion should first be understood as pathological phenotypes co-occurring with poor repair; they may contribute to a deteriorating repair environment or may be secondary to more severe primary injury (139). Distinguishing whether these changes are causes, consequences, or concomitant alterations still requires serial time-point observations and functional interventions for verification.

### 6.2 Chronic pain: bidirectional association rather than unidirectional causation

Chronic pain is a common clinical problem in patients after fractures and with nonunion, and its relationship with abnormal bone repair is more likely bidirectional than simply causal. Persistent inflammation, nerve injury, and local mechanical stimulation can induce peripheral sensitization, a process in which inflammatory mediators such as TNF-α, IL-1β, and prostaglandins, alongside neuropeptides such as SP and CGRP, contribute to pain persistence (140–142). In parallel, pain may indirectly affect functional recovery by limiting weight-bearing and rehabilitation activities, enhancing systemic stress, and maintaining neuronal sensitization (143–145). Mechanical instability, local inflammation, and unhealed tissue themselves can continuously generate nociceptive input. Thus, chronic pain and abnormal bone repair may influence each other and are jointly modulated by injury severity, infection, soft tissue damage, and mechanical stability.

Clinical longitudinal studies further support an association between persistent pain and poor recovery outcomes. Post-fracture pain can persist for 6–12 months or longer after injury and is associated with limited functional recovery, reduced activity and weight-bearing capacity, and decreased quality of life (146); in some patients with delayed healing or nonunion, a high pain burden can also persist long-term (147). Long-term follow-up studies also suggest that even after bony union is achieved, some patients may continue to experience significant pain and functional impairment, indicating that pain trajectories and radiographic healing are not fully synchronized (148). However, most current clinical studies primarily categorize pain as a patient-reported outcome or a concomitant phenotype of abnormal healing, and studies that longitudinally assess pain changes, radiographic callus formation, healing time, and nonunion outcomes within the same cohort remain limited (149). Therefore, existing evidence supports a clinical association between chronic pain and abnormal bone repair but is insufficient to demonstrate that pain itself can independently delay callus formation or cause nonunion.

Based on existing mechanistic and clinical evidence, the sequence of “pain-neuronal sensitization-persistent inflammation-sympathetic activation-impaired bone repair” should be regarded as a biologically plausible feedback loop rather than a definitive causal chain established by human longitudinal studies. Therefore, the most clearly defined clinical value of pain management currently remains symptom relief and facilitation of activity, weight-bearing, and functional rehabilitation; whether analgesia can directly accelerate fracture healing through neuro-immune mechanisms still lacks sufficient evidence. Future long-term prospective cohorts should synchronously record pain trajectories, analgesic exposure, activity and weight-bearing levels at multiple time points, combined with serial radiographic callus formation, healing time, nonunion, reoperation, and patient-reported outcomes, to clarify whether chronic pain is an early predictive marker, a potential contributing factor, or a consequence of impaired bone healing.

### 6.3 Dominant imbalance patterns under different pathological backgrounds

Diabetes, osteoporosis, aging, and infection all increase the risk of impaired bone repair, but their neuro-immune-bone abnormalities are distinct. Rather than treating these conditions as mutually independent examples, it is more appropriate to compare them across four dimensions: neural abnormalities, inflammatory patterns, vascular function, and bone cell responses.

In diabetes, prominent abnormalities involve the coexistence of peripheral neuropathy, microvascular damage, oxidative stress, and chronic low-grade inflammation (150). Sensory nerve dysfunction can be accompanied by altered CGRP and SP signaling, while the hyperglycemic environment can also affect macrophage function, BMSCs osteogenic potential, and endothelial cell responses (151). However, reduced CGRP or SP should not be viewed in isolation as a determinant of diabetic bone healing impairment, but rather as part of the combined effects of neuropathy, vascular dysfunction, and immune abnormalities.

The main features of osteoporosis are impaired baseline bone mass and microarchitecture, along with osteoblast-osteoclast coupling imbalance (152, 153). Sympathetic activity, chronic inflammation, and T cell-related signals may further regulate bone metabolism, but their relative contributions during fracture repair remain incompletely defined. Therefore, the more central issue in the osteoporotic context is low bone formation reserve and high bone resorption tendency, with neuro-immune abnormalities potentially acting as amplifying factors rather than independent initiating events.

Aging manifests as the simultaneous presence of immunosenescence, chronic low-grade inflammation, attenuated vascular responses, diminished neural regenerative capacity, and reduced osteoprogenitor function (154). Compared with diabetes, aging is more characterized by an overall decline in multi-system repair reserve rather than abnormality of a single neural or immune pathway. These changes can collectively delay inflammation resolution, neurovascular reconstruction, and callus maturation.

Infectious bone defects or post-traumatic osteomyelitis exhibit different features. PAMPs and DAMPs continuously stimulate innate immune responses, leading to high-intensity inflammation, tissue destruction, and bone resorption, with secondary damage to neural and vascular structures (155, 156). In this context, persistent inflammation usually has a more clearly defined upstream driving factor, whereas nerve injury and neuropeptide changes are more likely to be concomitant or amplifying components of infectious tissue damage and ongoing inflammation. Thus, although different pathological states can all manifest as neuro-immune-bone imbalance, their dominant mechanisms differ: diabetes by neuropathy-microvascular injury-metabolic inflammation; osteoporosis by low bone formation reserve and osteoblast-osteoclast imbalance; aging by a synchronized decline in multi-system repair capacity; and infection by persistent pathogen-associated inflammation and tissue destruction as the core driver. This comparison helps avoid simplistically reducing different pathological backgrounds to the same neuro-immune imbalance pattern.

### 6.4 From association networks to pathological feedback models

Synthesizing the above evidence, neural, immune, vascular, and bone cell changes in abnormal bone repair are more likely to constitute an interacting network rather than a fixed unidirectional pathological sequence. Some local mechanisms have relatively direct experimental support, such as inflammatory factors promoting sensory neuronal sensitization, persistent inflammation suppressing osteogenesis, and neural signals regulating immune cell function; however, the complete feedback chain of “insufficient reinnervation-persistent inflammation-sympathetic imbalance-vascular impairment-nonunion” currently remains an integrative pathological model. Therefore, the abnormal feedback loops proposed in this review must be categorized into two types of evidence: one consists of locally supported molecular or cellular links confirmed experimentally, and the other comprises higher-order feedback relationships integrated from these links but not yet comprehensively validated in the same fracture model. This evidentiary boundary is particularly important for interpreting CGRP, SP, and sympathetic signaling, as their effects are markedly time- and context-dependent. Future research needs to synchronously track reinnervation, neuropeptide changes, immune cell status, vascular reconstruction, and callus formation within the same model, combined with time-specific interventions, to definitively distinguish which abnormalities are driving factors of impaired bone healing, which are secondary consequences, and which merely represent co-occurring pathological phenotypes (**Figure 5**).

**Figure 5:**
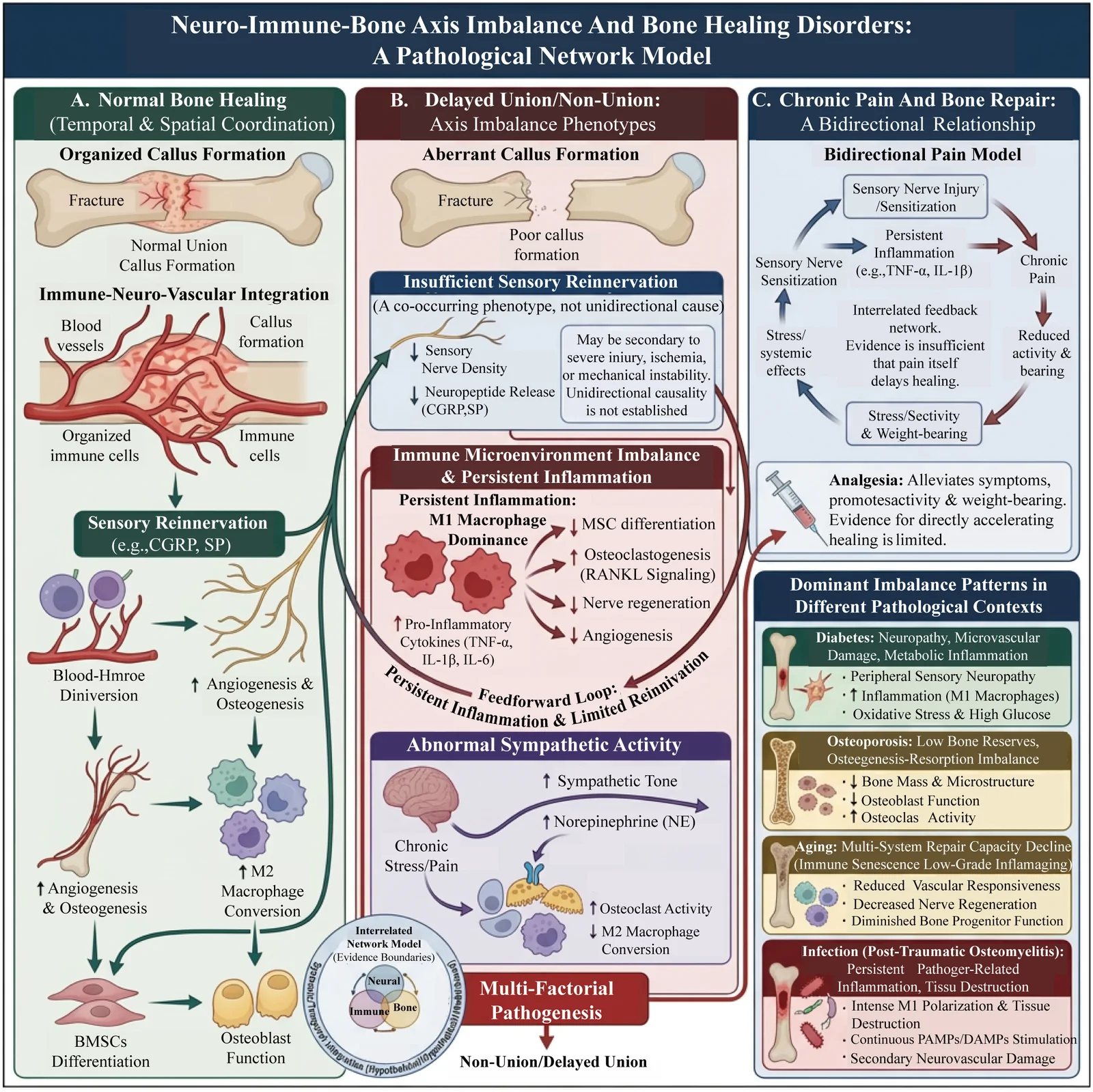
Pathological network model of neuro-immune-bone axis imbalance and impaired bone healing. This image outlines the differences in neural, immune, and bone repair networks between normal bone healing and delayed healing/nonunion. **(A)** Normal bone healing: orderly angiogenesis, immune cell recruitment, sensory nerve reinnervation, and release of neuropeptides such as CGRP/SP jointly promote BMSC differentiation, osteoblast function, and callus formation. **(B)** Delayed healing/nonunion: insufficient sensory nerve reinnervation, decreased neuropeptide release, persistent M1-like inflammation, enhanced RANKL-related osteoclast activity, suppressed MSC differentiation, and inadequate angiogenesis mutually amplify, forming a vicious cycle of “persistent inflammation-limited reinnervation”; abnormal sympathetic activity and elevated NE can also enhance osteoclast activity and suppress M2-like reparative responses. **(C)** Chronic pain and bone repair: bidirectional feedback exists among neuronal sensitization, persistent inflammation, and reduced activity and weight-bearing; analgesia facilitates the restoration of activity and weight-bearing, but evidence for its direct role in promoting callus formation remains limited. Different pathological backgrounds, such as diabetes, osteoporosis, aging, and infection, can generate distinct neuro-immune-bone axis imbalance patterns.

## 7 Therapeutic implications, translational challenges, and future directions

### 7.1 Translational principles and evidence stratification for targeting the neuro-immune-bone axis

The neuro-immune-bone axis offers a novel intervention perspective for post-traumatic bone regeneration. However, its clinical value lies not in simply enhancing a single neural signal or persistently suppressing inflammation, but in implementing temporally matched, localized, and controllable modulation based on the repair phase, pathological context, and local microenvironment. The early phase of fracture healing requires moderate inflammation and neurogenic signals for damage clearance and repair initiation; the intermediate phase depends on inflammation resolution, neurovascular reconstruction, and progressive establishment of osteogenic programs; the late phase is characterized primarily by osteoblast-osteoclast coupling and tissue remodeling (157, 158). Therefore, long-term or systemic interventions that lack precise temporal window selection may disrupt normal repair rhythms.

The translational maturity of existing therapeutic strategies varies considerably, and clinical applications, early translational efforts, and animal experimental results should not be equated in terms of evidentiary strength. Overall, they can be categorized into four tiers, namely: adjunctive therapies with some clinical application basis, such as certain electrical stimulation modalities and pain management; strategies with early translational potential but lacking sufficient fracture outcome evidence, such as local neuropeptide delivery and certain neuromodulation approaches; strategies involving macrophage function modulation, Treg-related strategies, EVs, and multifactor delivery, which are mainly at the preclinical stage; and complex cell engineering, genetic modification, and highly integrated neurovascularized scaffolds, which remain at the proof-of-concept stage. Therefore, evaluating any intervention strategy should simultaneously consider its primary target, mechanism of action, appropriate intervention phase, delivery method, level of evidence, and safety risks, rather than judging its clinical translational value solely based on osteogenic or anti-inflammatory effects.

### 7.2 Neuromodulation strategies: from clinical adjunctive therapy to localized targeted intervention

Currently, the neuromodulation approaches with greater translational significance encompass electrical stimulation, local neuropeptide delivery, sympathetic nervous system modulation, and pain management, but the maturity of evidence varies significantly among these strategies. Electrical stimulation is the direction with relatively more substantial clinical foundation. Certain bone growth electrical stimulation techniques have been used as adjunctive therapy for delayed union or nonunion, with potential effects involving local electrophysiological environments, cell proliferation, blood flow, and regulation of osteogenesis-related signals (159–161). However, the mechanisms of action and indications of different stimulation modes are not entirely consistent, and their analgesic effects cannot be equated with bone healing-promoting effects. Therefore, it is currently more suitable as adjunctive therapy for specific patients rather than a universally applicable neuro-immune modulation strategy.

Local delivery of neuropeptides such as CGRP is mainly in the preclinical or early translational stage. Animal and bone repair models suggest that CGRP can support angiogenesis and osteogenic responses (162). Given CGRP’s limited half-life and therapeutic window, achieving localized, short-range, and controlled delivery using hydrogels, nanocarriers, or degradable scaffolds may be more translationally viable than systemic continuous exposure. Meanwhile, attention should also be paid to vasodilation, sensory sensitization, and other off-target effects, avoiding direct extrapolation of experimental pro-repair effects to clinical benefits.

Sympathetic modulation also holds mechanistic appeal. Sustained β-adrenergic signaling is associated with suppressed osteogenesis, enhanced bone resorption, and altered immune status (163), but β-blockers as bone healing-promoting drugs still lack sufficient randomized clinical evidence. In particular, systemic sympathetic modulation may cause alterations in heart rate, blood pressure, and other cardiovascular effects; therefore, its potential bone metabolic benefits must be evaluated concurrently with systemic safety.

Pain management should be strictly distinguished from bone regeneration-promoting therapy. Clinically, adequate analgesia can improve patient comfort and may indirectly support fracture recovery by promoting activity, weight-bearing, and functional rehabilitation; however, there is currently insufficient evidence to demonstrate that pain relief itself directly promotes callus formation or shortens fracture healing time through neuro-immune mechanisms (164, 165). Furthermore, different analgesic drugs may have varying effects on inflammatory responses, bone metabolism, and fracture repair; hence, analgesic efficacy cannot serve as a surrogate endpoint for bone regeneration benefits. Future research should separately evaluate pain trajectories, activity and weight-bearing recovery, patient-reported outcomes, and radiological bone healing to distinguish the three levels of therapeutic effects: pain relief, functional improvement, and biological bone healing. In contrast, neurotrophic factors such as NGF and BDNF currently still hold mainly mechanistic research value, with limited direct clinical translational evidence in bone repair; particularly, NGF may enhance nociception and pain sensitization, making its therapeutic window and safety profile more complex. Therefore, these neurotrophic signals are currently more suitable as mechanistic targets and preclinical research directions rather than mature bone regeneration interventions.

### 7.3 Immunomodulation strategies: restoring inflammatory timing rather than sustained anti-inflammation

The core goal of immunological intervention is to restore the dynamic transition among inflammation initiation, inflammation resolution, and tissue repair, rather than chronically suppressing inflammatory levels. Currently, the more prominent directions mainly include macrophage function modulation, Treg-related interventions, and targeting of pro-inflammatory cytokines, but overall, evidence remains predominantly preclinical.

Macrophage function modulation has a strong mechanistic basis. Signals such as IL-4, IL-13, and IL-10 can enhance inflammation resolution, angiogenesis, and tissue repair-related functions in experimental models (166, 167). However, macrophages *in vivo* exhibit high heterogeneity and plasticity; therefore, the therapeutic goal should not be simply defined as inducing fixed “M2 polarization,” but rather promoting functional states that match specific repair phases. Systemic cytokine therapy may also cause immunosuppression, increased susceptibility to infection, and off-target effects; thus, local, low-dose, and short-term interventions may be more feasible.

Treg-related modulation also has potential value. Tregs can limit excessive inflammation through signals such as IL-10 and TGF-β (168, 169), but direct causal evidence in traumatic fractures remains limited. Direct cell therapy also faces challenges including complex manufacturing, insufficient stability, high costs, and systemic immunosuppression. In contrast, moderately promoting endogenous Treg recruitment and function through the local microenvironment or material platforms may have greater translational potential, but the optimal time window and infection risks still need further clarification.

Therapies targeting inflammatory cytokines such as TNF-α and IL-1β particularly need to consider the repair phase. Sustained high-level inflammation can impair BMSCs, vascular responses, and osteogenic reactions, but these factors also participate in tissue clearance and immune cell recruitment during the early injury phase (170). Premature or excessive immunosuppression may delay early repair and impair anti-infection capacity; therefore, broad and sustained anti-inflammatory therapy is currently not supported as a routine bone healing-promoting strategy. Future efforts should focus more on identifying aberrant persistent inflammation and its appropriate intervention window, rather than suppressing physiological early inflammatory responses.

### 7.4 Biomaterials and delivery platforms: achieving localized and staged multi-target regulation

The core value of biomaterials in the neuro-immune-bone axis lies not only in their role as carriers, but also in their ability to integrate local delivery, temporal control, and multi-cellular regulation within a single therapeutic platform. Hydrogels, nanocarriers, and degradable scaffolds can enhance the local retention of CGRP, cytokines, VEGF, bone morphogenetic proteins, EVs, or small-molecule drugs at the fracture site while reducing systemic exposure (171). Beyond delivery functions, bone repair materials also need to balance local mechanical support and osteogenic activity. For example, recently developed injectable carbon nanotube-reinforced calcium phosphate bone cement can improve material mechanical properties and promote BMSCs osteogenic differentiation and bone regeneration, suggesting that material structural performance and bioactivity can be synergistically optimized within the same platform (172). More importantly, material platforms can achieve staged release aligned with the temporal dynamics of repair. For instance, early stages may focus on inflammation modulation and vascular environment improvement, intermediate stages on enhancing neurovascular reconstruction and osteogenic support, and late stages on promoting callus maturation and tissue remodeling (173). Certain conductive, piezoelectric, and stimuli-responsive materials can also combine physical stimulation with molecular delivery, offering possibilities for multidimensional microenvironmental regulation (174). However, the more complex the material system’s functions, the greater the challenges in manufacturing consistency, management of degradation products, biocompatibility, sterilization, quality control, and regulatory oversight. EVs similarly face issues such as source heterogeneity, unclear active components, dosage standardization, and batch-to-batch consistency (175). Cell-loaded scaffolds, genetically modified cells, and complex 3D-bioprinted neuro-vascularized constructs should currently remain positioned mainly at the preclinical or proof-of-concept stage (176). Therefore, the criteria for evaluating future material strategies should shift from “whether the functions are sufficiently complex” to “whether effective intervention can be achieved for specific bone repair problems in a manufacturable, controllable, and regulatory-compliant manner”.

### 7.5 Clinical translation challenges and future research priorities

The clinical translation of therapies targeting the neuro-immune-bone axis still faces multiple challenges, with the most critical issue being how to identify regulatory nodes with true causal significance and implement safe, effective interventions at appropriate time windows and in suitable patient populations. The effects of many neuro-immune targets vary with the repair phase; therefore, future research needs to determine not only “what to target” but also “when to intervene” and “how long to sustain”. Technologies such as single-cell sequencing, time-series transcriptomics, and intravital imaging can be used to delineate cellular states and signaling changes across different repair phases, providing a basis for identifying key therapeutic time windows.

Furthermore, distinguishing between direct neural effects and those mediated indirectly by immune or vascular mechanisms remains a significant challenge. The bone repair effects observed for CGRP, SP, and NE may arise from direct actions on BMSCs, osteocytes, or endothelial cells, or may first alter macrophages, T cells, and the vascular microenvironment, subsequently indirectly influencing osteogenesis and bone remodeling. Spatial omics, multiplexed tissue imaging combined with neural tracing, cell-specific receptor interventions, and time-specific functional experiments offer promising avenues for elucidating the true signaling directions and relative contributions among different cells. Such technologies can also be used to test whether the “neuro-vascular-immune-bone regeneration niche” proposed in this review possesses a stable spatial and functional basis-that is, whether different cells form identifiable spatial neighborhoods within the callus, and whether disruption or restoration of this structure can alter bone healing outcomes.

Another key issue is distinguishing driving factors from secondary changes in aberrant healing. Although impaired reinnervation, persistent inflammation, and vascular dysfunction often coexist with delayed union or nonunion, it remains difficult to determine whether they are early causative factors or consequences of severe tissue damage, mechanical instability, or persistent non-healing. Longitudinal imaging, serial biomarker monitoring, and local tissue multi-omics can help establish the temporal sequence between these changes and callus formation, while time-specific functional interventions are essential for further validating causal relationships. Only by clarifying which abnormalities truly drive healing failure can we avoid mistaking accompanying biomarkers for therapeutic targets.

The gap between existing experimental systems and clinical complex fractures also limits the extrapolation of candidate strategies. Murine models of bone defects, denervation, diabetes, or infection cannot fully recapitulate the mechanical instability, severe soft tissue damage, aging, comorbidities, and long-term drug exposure commonly seen in clinical patients (177, 178).

Therefore, before advancing to clinical trials, candidate therapies should be further validated in weight-bearing long bone fractures, large animal models, and multi-comorbidity models, with concurrent evaluation of long-term safety. Particular attention should be paid to the impact of sustained immunosuppression on infection control and early repair, the cardiovascular risks of systemic sympathetic modulation, and the sensory, vascular, and other off-target effects that neuropeptides and neurotrophic factors may cause. For multi-system interventions such as those targeting the neuro-immune-bone axis, safety evaluation should be given equal importance to bone healing-promoting efficacy.

Patient heterogeneity further increases the complexity of clinical translation. Diabetic patients may exhibit more prominent neuropathy, microvascular damage, and metabolic inflammation; elderly patients more often present with immunosenescence, reduced neural regeneration capacity, and diminished overall repair reserve; and patients with infected bone defects typically present with persistent pathogen-associated inflammation and tissue destruction. These differences suggest that a unified “axis modulation” regimen applicable to all patients is unlikely to exist. Establishing dynamic phenotyping systems that integrate imaging data, circulating biomarkers, and inflammatory and metabolic profiles, and using them to identify dominant imbalance patterns and potential therapeutic targets in different patients may be an important prerequisite for achieving precision intervention.

Future technological development should therefore not merely focus on adding new cell subsets, molecular targets, or material platforms, but should revolve around several unresolved clinical questions: which cells undergo critical interactions, where and when; which neuro-immune signals are true causal drivers; which abnormalities can serve as early predictive markers and therapeutic targets; which patients are most likely to benefit from specific interventions; and what treatments can improve bone healing within an acceptable safety margin. Only by further answering “when to intervene, what to target, and for whom,” can the neuro-immune-bone axis gradually evolve from an integrative mechanistic framework into a verifiable precision therapeutic strategy (**Table 4**).

**Table 4:** Intervention strategies, evidence levels, and translational stages targeting the neuro-immune-bone axis to promote post-traumatic bone regeneration.

Intervention direction	Representative strategy/target	Main therapeutic rationale	Main evidence source/level of evidence	Translational stage	Key translational limitations and safety	Reference
Electrical stimulation and neuromodulation	Bone growth electrical stimulation, certain transcutaneous electrical stimulation modalities.	Modulate the local electrophysiological environment, blood flow, and osteogenesis-related signals, and may improve functional recovery.	Clinical evidence as adjunctive therapy; some techniques have been used for delayed union or nonunion, but evidence for bone healing across different stimulation modalities is inconsistent.	Clinical adjunctive application	It is necessary to clarify stimulation modality, intensity, duration, fracture type, and target populations; analgesic effects cannot be equated with pro-osteogenic effects.	(159–161)
CGRP-related local neuromodulation	Local CGRP supplementation, hydrogels, nanocarriers, or biodegradable scaffold delivery.	Supports angiogenesis and osteogenic responses, and may modulate the local reparative microenvironment.	Predominantly preclinical evidence in fracture/bone repair.	Preclinical-early translation	Limited half-life and therapeutic window; requires local, short-range, controlled release, and assessment of vasodilation, sensory sensitization, and off-target effects.	(162)
Sympathetic/β-adrenergic modulation	β-blockade, sympathetic signaling modulation.	Mitigates osteogenic suppression, enhanced bone resorption, and immune dysregulation associated with sustained sympathetic input.	Primarily animal, bone metabolism studies, and human observational evidence; lacks adequate fracture intervention trials.	Early translation; pro-bone healing indication not yet established.	Systemic modulation may affect heart rate and blood pressure; need to clarify local versus systemic sympathetic effects, suitable population, and cardiovascular safety margins.	(163)
Pain management	Pharmacological analgesia and other clinical pain control measures.	Improves comfort and indirectly supports functional recovery by promoting activity, weight-bearing, and rehabilitation.	Relatively sufficient clinical evidence, but mainly supports analgesia and functional benefits, rather than directly promoting callus formation.	Routine clinical supportive care.	Pain relief cannot serve as a surrogate endpoint for bone regeneration benefit; the effects of different analgesics on inflammation and bone metabolism need separate evaluation.	(164, 165)
Neurotrophic factor modulation	NGF, BDNF, etc.	May support nerve regeneration and neuro-vascular-related repair processes.	Predominantly mechanistic studies and preclinical evidence.	Preclinical/concept validation	Limited fracture-specific efficacy evidence; NGF may enhance nociception and pain sensitization.	(164, 165)
Macrophage functional modulation	IL-4, IL-13, IL-10, and material-mediated local immunomodulation.	Promotes inflammation resolution, angiogenesis, and macrophage functional states matched to tissue repair.	Substantial preclinical experimental evidence, but insufficient human fracture intervention evidence.	Preclinical	Therapeutic strategies should not aim solely for fixed “M2 polarization”; systemic administration may increase immunosuppression and infection risk.	(166, 167)
Treg-related immunomodul-ation	Treg recruitment, IL-10/TGF-β-related modulation, local microenvironment induction.	Limits persistent inflammation and improves the repair-related immune environment.	General immunology and preclinical bone repair evidence; limited fracture-specific causal evidence.	Preclinical	Cell therapy faces challenges in manufacturing, stability, cost, and immunosuppression risk.	(168, 169)
Phasic modulation of pro-inflammatory factors	TNF-α, IL-1β, and other inflammatory pathways.	Reduces tissue damage and improves the osteogenic microenvironment in the context of abnormal persistent inflammation.	Predominantly preclinical and indirect mechanistic evidence.	Preclinical; routine pro-bone healing application not yet supported	Premature or sustained inhibition may interfere with necessary early inflammation and increase infection risk.	(170)
Biomaterials and local controlled delivery	Hydrogels, nanocarriers, biodegradable scaffolds, CGRP/VEGF/BMP/EVs, etc.	Improves local retention, reduces systemic exposure, and enables temporally controlled release.	Abundant preclinical bone repair research; complex neuro-immune multi-target systems remain largely experimental.	Preclinical-early translation	Need to optimize loading, release kinetics, degradation behavior, and address manufacturing consistency, biosafety, sterilization, and regulatory issues.	(171, 173)
Mechano-bioactive integrated materials	Injectable carbon nanotube-reinforced calcium phosphate bone cement, etc.	Simultaneously improves local mechanical support and osteogenic activity.	Preclinical evidence for bone regeneration.	Preclinical	Need further evaluation of long-term safety, degradation behavior, weight-bearing performance, and clinical manufacturability.	(172)
Complex cell/gene and neurovascularized constructs	Cell-loaded scaffolds, genetically modified cells, complex 3D bioprinted neuro-vascularized scaffolds.	Attempts to simultaneously reconstruct neural, vascular, immune, and osteogenic microenvironments.	Engineering and preclinical concept validation evidence.	Concept validation/early preclinical	Complex manufacturing, difficult batch-to-batch consistency and quality control, and long-term safety, cost, and regulatory barriers.	(176)

## 8 Conclusion

Existing evidence supports the conceptualization of neural, immune, vascular, and bone repair processes as an interconnected regulatory network in post-traumatic bone regeneration. The temporal coordination among nerve reinnervation, immune state transition, and neurovascular remodeling may influence callus formation and remodeling. However, the “nerve-immune-bone axis” is currently more suitable as a working framework for integrating existing evidence rather than an established unified causal pathway. Notably, neural, immune, and vascular abnormalities in delayed healing and nonunion may either contribute to repair failure or be secondary consequences of severe tissue damage, infection, mechanical instability, or persistent nonunion. A critical evidence gap remains, as many proposed mechanisms are derived primarily from *in vitro* studies, bone defect models, denervation protocols, or inflammation and pain models, rather than from traumatic fracture models capable of simultaneously establishing temporal sequence, spatial relationships, and functional causality. Future research should prioritize clarifying key nerve-immune cell interactions at different repair stages, distinguishing direct neural effects from immune- or vascular-mediated indirect effects, and verifying whether concepts such as the “nerve-vascular-immune-bone regeneration niche” have a genuine spatial and functional basis. At the translational level, efforts must integrate more clinically representative fracture models, longitudinal human studies, and patient stratification to systematically determine intervention time windows, appropriate targets, local delivery methods, safety boundaries, and the populations most likely to benefit. Resolving these issues will determine whether the nerve-immune-bone axis can evolve from a mechanistic framework into a verifiable precision bone regeneration strategy.
